# Tumoroid Model Reveals Synergistic Impairment of Metabolism by Iron Chelators and Temozolomide in Chemo‐Resistant Patient‐derived Glioblastoma Cells

**DOI:** 10.1002/advs.202412505

**Published:** 2025-04-26

**Authors:** Meitham Amereh, Amir Seyfoori, Shahla Shojaei, Sarah Lane, Tian Zhao, Mahdieh Shokrollahi Barough, Julian J. Lum, Patrick Walter, Mohsen Akbari

**Affiliations:** ^1^ Laboratory for Innovations in Micro Engineering (LiME) Department of Mechanical Engineering University of Victoria Victoria BC V8P 5C2 Canada; ^2^ Department of Human Anatomy and Cell Science Max Rady College of Medicine Rady Faculty of Health Sciences University of Manitoba Winnipeg MB R3T 2N2 Canada; ^3^ Department of Biology University of Victoria BC Canada; ^4^ Trev and Joyce Deeley Research Centre BC Cancer Victoria BC V8R 6V5 Canada; ^5^ Department of Biochemistry and Microbiology University of Victoria Victoria BC V8W 2Y2 Canada; ^6^ Terasaki Institute for Biomedical Innovations Los Angeles CA 91367 USA

**Keywords:** chemoresistance, glioblastoma, iron metabolism, metabolic sensitivity, tumoroid model

## Abstract

Chemoresistance poses a significant clinical challenge in managing glioblastoma (GBM), limiting the long‐term success of traditional treatments. Here, a 3D tumoroid model is used to investigate the metabolic sensitivity of temozolomide (TMZ)‐resistant GBM cells to iron chelation by deferoxamine (DFO) and deferiprone (DFP). This work shows that TMZ‐resistant GBM cells acquire stem‐like characteristics, higher intracellular iron levels, higher expression of aconitase, and elevated reliance on oxidative phosphorylation and proteins associated with iron metabolism. Using a microphysiological model of GBM‐on‐a‐chip consisting of extracellular matrix (ECM)‐incorporated tumoroids, this work demonstrates that the combination of iron chelators with TMZ induces a synergistic effect on an in vitro tumoroid model of newly diagnosed and recurrent chemo‐resistant patient‐derived GBM and reduced their size and invasion. Investigating downstream metabolic variations reveal reduced intracellular iron, increased reactive oxygen species (ROS), upregulated hypoxia‐inducible factor‐1α, reduced viability, increased autophagy, upregulated ribonucleotide reductase (RRM2), arrested proliferation, and induced cell death in normoxic TMZ‐resistant cells. Hypoxic cells, while showing similar results, display reduced responses to iron deficiency, less blebbing, and an induced autophagic flux, suggesting an adaptive mechanism associated with hypoxia. These findings show that co‐treatment with iron chelators and TMZ induces a synergistic effect, making this combination a promising GBM therapy.

## Graphical abstract

This study investigates the metabolic vulnerabilities of glioblastoma (GBM), an aggressive brain tumor. Research demonstrates that iron chelation therapy, in combination with standard chemotherapy, significantly enhances the efficacy of treatment against resistant GBM cells in a 3D tumoroid model.

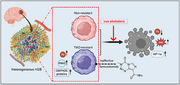

## 1 Introduction

Glioblastoma (GBM) is among the most malignant glial tumors with a low survival rate of 5.5% of patients living 5 years post‐diagnosis.^[^ ^1^, ^2^ ^]^ In identifying contributing factors to this low survival rate, it has become clear that there is heterogeneity in the mutations that play an important role in the pathology of GBM. Ninety percent of GBMs are primary, but there are secondary as well, and understanding the mutations of both helps to understand the molecular architecture of GBM. The following genetic alterations are common to both primary and secondary GBMs: amplification or mutation of epidermal growth factor receptor (EGFR), kinase inhibitor 2 A/B (CDKN2A/B) deletion, tumor protein 53 (TP53) mutation, platelet‐derived growth factor receptor (PDGFR) gene amplification, cyclin‐dependent, phosphatase and tensin homolog (PTEN), isocitrate dehydrogenase 1/2 (IDH1/2) mutation, and promoter methylation of O6‐methylguanine‐DNA methyltransferase (MGMT).^[^ ^3^ ^]^ Conventional treatment of GBM uses temozolomide (TMZ), an alkylating agent that targets guanine, causing the insertion of thymine instead of cytosine opposite the methylguanine during subsequent DNA replication, which ultimately leads to apoptosis.^[^ ^4^ ^]^ The limited effectiveness of TMZ in GBM treatment is attributed to some of the genetic alterations known to GBM, which include downregulation of MGMT, mutated P53, overexpression of anti‐apoptotic protein Myeloid leukemia 1 (MCL‐1), and the presence of tumor residues containing resistant cancer stem cell (CSC) populations.^[^ ^5^, ^6^, ^7^ ^]^ These genetic alterations allow the TMZ‐resistant cells to increase nutrient acquisition, such as iron that sustains their robust growth potential. In particular, TMZ‐resistant cells also exhibit distinct alterations, including more stem cell like behavior and modifications of cell metabolism when compared to their non‐resistant counterparts, including significant variations in glucose uptake, iron utilization, and responses to hypoxia within the tumor microenvironment (TME).^[^ ^8^ ^]^ These metabolic deviations, which enhance energy production and growth potential, also aid in the preservation of drug resistance and tumor lifetime.^[^ ^9^ ^]^

The hypoxic microenvironment in tumors contributes to chemo‐resistance by inducing adaptive responses and altering cellular metabolism, reducing drug efficacy and treatment resistance in these aggressive cancer cells.^[^ ^10^ ^]^ Non‐physiological oxygen levels in hypoxia alter the metabolism of cancer cells and induce epithelial‐to‐mesenchymal transition (EMT), angiogenesis, upregulate proinflammatory cytokines, etc., which in turn contribute to therapy resistance.^[^ ^11^ ^]^ hypoxia‐inducible factor‐1α (HIF‐1α) is a heterodimeric partner with hypoxia inducible factor‐1β (HIF‐1β) of the DNA binding transcription factor complex to activate the hypoxia gene.^[^ ^12^ ^]^ In general, the oxygen levels in different types of hypoxic tumors range from 0.3–4.2%, where the median for brain tumors is ≈1.7%.^[^ ^13^, ^14^, ^15^ ^]^ Under hypoxic or iron‐depleted conditions, hydroxylation reactions are inhibited, leading to rapid HIF‐1α accumulation.^[^ ^16^ ^]^ Hypoxia also has a close and complex link with autophagy. Autophagy can regulate homeostasis of hypoxia‐stressed cancer cell by producing adenosine triphosphate (ATP) via recycling free amine and fatty acids.^[^ ^17^ ^]^ Additionally, hypoxia notably exerts a direct influence on basal glucose uptake in mammalian cells, where cell growth and survival become highly dependent on glucose concentration.^[^ ^18^ ^]^ This influence is magnified in a tumor where its 3D structure imposes nonuniform glucose and oxygen distributions due to diffusion.^[^ ^19^ ^]^ In addition, studies have shown that autophagy plays dual roles of anti/pro‐survival in TMZ‐resistant GBM's response to chemotherapy and radiotherapy.^[^ ^20^ ^]^ The modulation of the autophagy pathway has been receiving attention as a new therapeutic approach in chemo‐resistant GBM.^[^ ^21^ ^]^ The exact mechanism of resistance remains unclear, making it a major focus of research in the field to understand the underlying mechanisms and identify novel therapeutic modalities.^[^ ^22^ ^]^ One of the pro‐survival interacting pathways in cancer cells is associated with iron metabolism which is regulated by consumption of glucose and oxygen level in cytoplasm.

Iron metabolism plays a significant role in promoting chemoresistance, as resistant cells can increase cytosolic iron availability to foster tumor growth and evade chemotherapy. Having enough iron is important to any cell as it facilitates oxidative phosphorylation, detoxification processes, ribosomal biogenesis, DNA replication, and repair.^[^ ^23^ ^]^ It is useful because of its capacity to redox cycle between iron (II) and (III), for example, many enzymes use iron as a cofactor in the form of iron‐sulfur clusters, heme‐iron, and mono‐iron and di‐iron. Important to any proliferating cell is its capacity to maintain the pool of deoxynucleotide triphosphates, facilitated by ribonucleotide reductase (RNR), which utilizes a di‐iron‐tyrosyl radical. Due to its redox cycling capacity, iron can also catalyze the formation of reactive oxygen species (ROS). In the Fenton reaction, reduced iron can catalyze the reduction of hydrogen peroxide to hydroxyl radicals that can facilitate lipid peroxidation inducing cell toxicity in a unique mechanism known as ferroptosis.^[^ ^24^ ^]^ Therefore, iron can act as both a pro‐survival and anti‐survival factor by participating in reactions that promote or inhibit DNA synthesis and ROS generation.

Iron chelators can sequester from the cell, depriving cancer cells of this essential element, which can potentially reduce their viability and proliferation.^[^ ^25^, ^26^ ^]^ In certain instances, using low doses, they can also change the basal ROS level in cells by decreasing ROS generation through iron sequestration, preventing its participation in the Fenton reaction.^[^ ^8^ ^]^ In contrast, iron chelators have been shown to increase ROS generation in different ways such as by exerting prooxidant effects, binding cellular iron, downregulating DNA synthesis, and increasing the labile iron pool (LIP).^[^ ^27^, ^28^, ^29^, ^30^ ^]^ As the basal level of ROS in cancer cells is higher than normal cells, they are more sensitive to upregulation of ROS. Such effects have been attracting attention, and iron chelating drugs are being explored as potential therapeutic drugs for cancer therapy.^[^ ^31^, ^32^ ^]^ Because they upregulate the expression of HIF1‐α and have a short circulating half‐life, they have tumor suppressing effects by inducing iron depletion. Deferoxamine (DFO) and deferiprone (DFP) are FDA‐approved iron chelators commonly used to alleviate systemic iron burden in thalassemia, and are in clinical trials to reduce iron overload in Alzheimer's (AD) and Parkinson's (PD) diseases.^[^ ^33^, ^34^ ^]^ Both DFO, widely known for its efficacy in iron binding, and DFP, a lipophilic chelator that can pass through the cell membrane and the blood‐brain barrier, were used in our study to gain a comprehensive understanding of their distinct contributions to iron homeostasis and their cytotoxic effects on GBM in our experimental model.

In this study, we employed a 3D tumoroid‐on‐a‐chip model of GBM to investigate the sensitivity of patient‐derived and immortalized GBM tumoroids to iron chelators. Utilizing this model, we recapitulated the physiologically relevant conditions of the tumor microenvironment (TME) and analyzed the impact of iron depletion on the viability, growth, and invasion of both non‐resistant and TMZ‐resistant tumoroids. We evaluated the antitumor effects of iron and oxygen deficiency on U251 GBM cells, using DFO and DFP, with the aim of relieving TMZ resistance. Using proteomics, we first characterized the molecular signature of TMZ‐resistant versus non‐resistant GBM in 2D and 3D cultures (Figure S1, Supporting Information). Furthermore, variations in several downstream components such as viability, proliferation, autophagy, intracellular iron content, ribonucleotide reductase (RNR) expression, apoptosis, and ROS were monitored in TMZ‐resistant versus non‐resistant GBM, and the capability of iron‐chelators to change metabolism and to induce cell death was obtained (see the schematic Figure S9, Supporting Information).

## 2 Results

### 2.1 TMZ‐Resistant Cells Acquired Characteristics of Cancer Stem Cells and Mitochondrial Respiration

**Figure 1:**
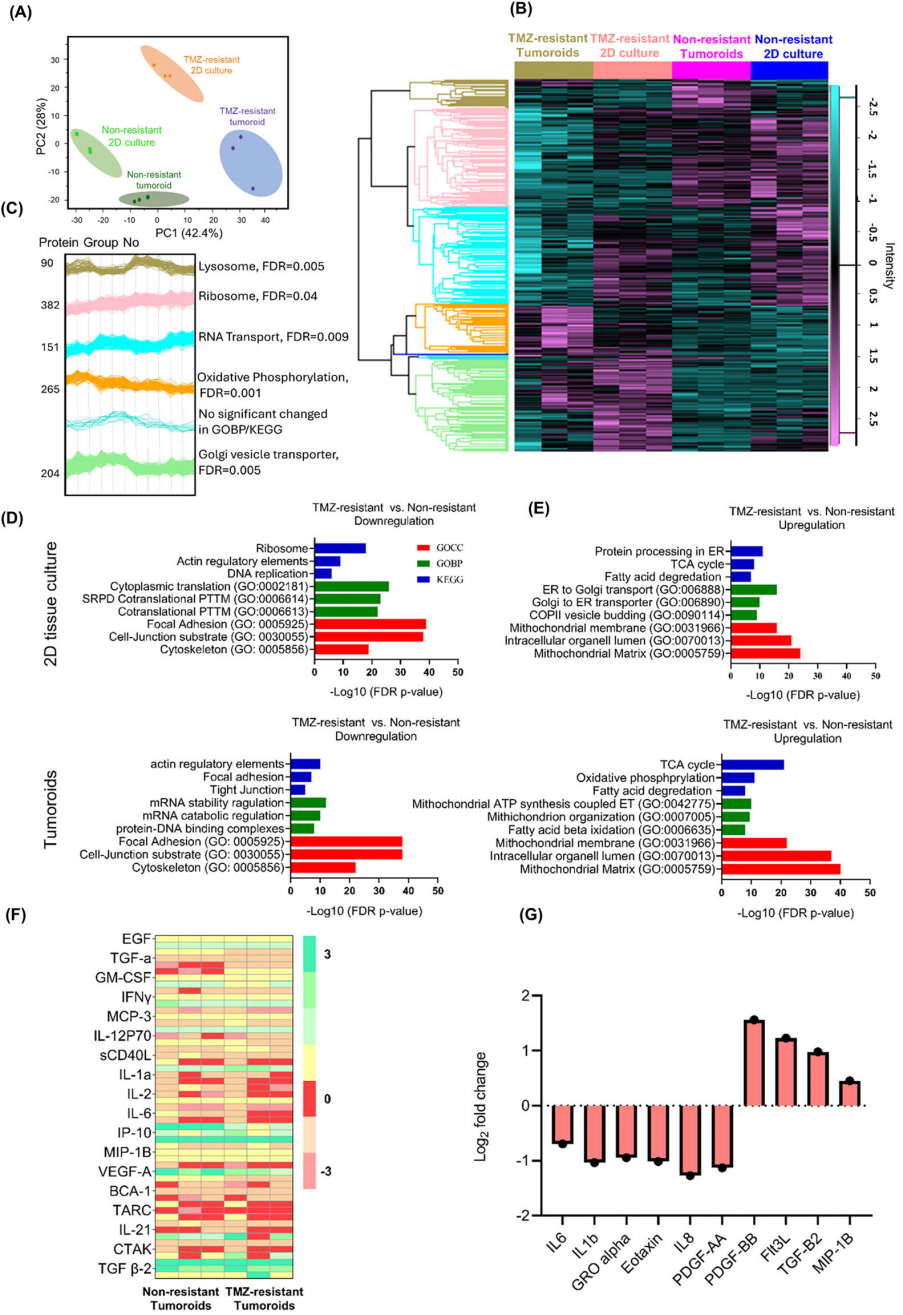
Global characterization and molecular signature of U251 non‐resistant and resistant in 2D and 3D models. A) Principal component analysis demonstrates a clear distinction between the proteomes of 2D tissue culture and 3D tumoroid in non‐resistant and TMZ‐resistant samples. B) Hierarchical clustering of normalized protein concentrations. Each raw represents a distinct protein and each column represents a sample (N = 3 biological independent experiment and one‐way ANOVA: Benjamini–Hochberg FDR = 0.05). C) Protein expression profiles for each cluster and the most enriched KEGG/GOBP per cluster is shown on the right side of each profile. D,E) The three most significant GO terms and KEGG pathways that upregulated or downregulated in each group emerged from the enrichment analysis of the genes identified by student *t*‐test. F) Hierarchical clustering of normalized cytokine concentrations. Each raw represents a distinct cytokine and each column represents a sample (significant cytokine was extracted based on the difference in their expression values with log2 fold change ≥ 2, FDR = 0.001). G) Cytokine profile comparing non‐resistant and TMZ‐resistant tumoroids, N = 3 biological independent experiment.

Molecular signatures of non‐resistant versus TMZ‐resistant cells were characterized via comprehensive proteomics and secretome analyses. Principal component (PC) analysis revealed differences between both TMZ‐resistant and non‐resistant cells, and between 2D and 3D culturing methods. PC1 and PC2 were sufficient to separate these conditions, accounting for 42% and 28% of the total variance, respectively (**Figure** **1**A). Hierarchical clustering identified 1100 differentially regulated proteins that corresponded to 3D culture and resistance characteristics (Figure 1B). The enrichment analysis revealed that acquiring resistance was linked to reduced expression of ribosomal and lysosomal and proteins, and increased expression of genes related to oxidative phosphorylation and vesicular transport (Figure 1C). Additionally, TMZ‐resistant cells in 2D and tumoroid cultures exhibited significant downregulation in cytoskeleton related protein and essential cell‐cell junction elements (*p* < 0.05). Some interesting proteins were differentially expressed in TMZ‐resistant cells (Volcano plot analysis, *p* < 0.05). These included upregulation of proteins associated with iron metabolism, such as four and sevenfold increase in ferritin light chain (FTL) and ferritin heavy chain (FTH1), and with regulation of cellular metabolism, such as a sixfold decrease in argininosuccinate synthase 1 (*p* < 0.05). TMZ‐resistant cells/tumoroids also upregulated proteins associated to citric acid cycle (TCA) compared to their non‐resistant counterparts. Cathepsin L1 (CTSL), a lysosomal cysteine protease enzyme, significantly decreased (threefold), suggesting a reduction in lysosomal protein expression in the context of 3D cultures (*p* < 0.05).

In 3D tumoroids, genes corresponding to ATP synthesis and fatty acid beta‐oxidation were enriched, while actin cytoskeleton regulation, focal adhesion, and tight junctions were downregulated (Figure 1D,E). TMZ‐resistant tumoroids exhibited the highest expression of proteins associated to oxidative phosphorylation (Figure 1B,C). We observed higher expression of aconitase.^[^ ^35^ ^]^

To understand the impact of TME on resistance characteristics, we studied the secretome profile of 68 cytokines from the inflammatory and invasion family. Among these, 18 cytokines were significantly altered (Figure 1F). The levels of hypoxia‐related cytokines, interferon gamma‐induced protein 10 (IP10) (2.2‐ and 2.0‐fold), vascular endothelial growth factor α (VEGF‐α) (4.1‐ and 4.8‐fold), growth‐regulated oncogen α (GRO‐α) (2.8‐ and 2.2‐fold), macrophage‐derived chemokine (MDC) (2.5‐ and 2.4‐fold), IL‐1RA (2.7‐ and 2.8‐fold), interleukin 8 (IL‐8) (6.7‐ and 2.2‐fold), monocyte chemoattractant protein‐1 (MCP1) (7.0‐ and 5.5‐fold), IL‐23 (2.6‐ and 2.5‐fold), transforming growth factor β1 (TGF‐β1) (7.2‐ and 7.0‐fold), and TGF‐β2 (4.1‐ and 4.0‐fold) were found to be higher in both non‐resistant and TMZ‐resistant tumoroids, respectively, compared to their counterparts grown in 2D culture. However, in TMZ‐resistant tumoroids, the levels of cytokines integral to the immune system such as TGF‐α, IP10, IL21, IL‐8, IL‐18, and platelet‐derived growth factor‐AA (PDGF‐AA) were decreased by more than twofold compared to non‐resistant tumoroids (Figure 1G). These results support the reduced inflammatory reactions identified in proteomic data of TMZ‐resistant tumoroids.

Overall, TMZ‐resistance was characterized by increased expression of genes related to cell survival, the TCA cycle, and oxidative phosphorylation, while decreased expression of genes related to cell differentiation, cellular connections, and inflammatory reactions. The elevated reliance of TMZ‐resistant cells to iron and oxygen for their metabolism and survival reveals their metabolic vulnerabilities. Additionally, these findings underscore the significant differences between monolayer and 3D culture conditions on cell phenotype in vitro, leading us to conduct 3D tumoroid investigations in addition to monolayer studies.

### 2.2 Cotreatment with Iron Chelators and TMZ Induced Synergic Effects on hGBM Tumoroids

Despite extensive efforts to develop new treatments for GBM, most drugs have not succeeded in improving patient survival. Poor clinical outcomes are mainly due to the failure of current preclinical models to accurately replicate the pathophysiology of GBM, including CSCs population and the 3D nature of TME.^[^ ^36^ ^]^

**Figure 2:**
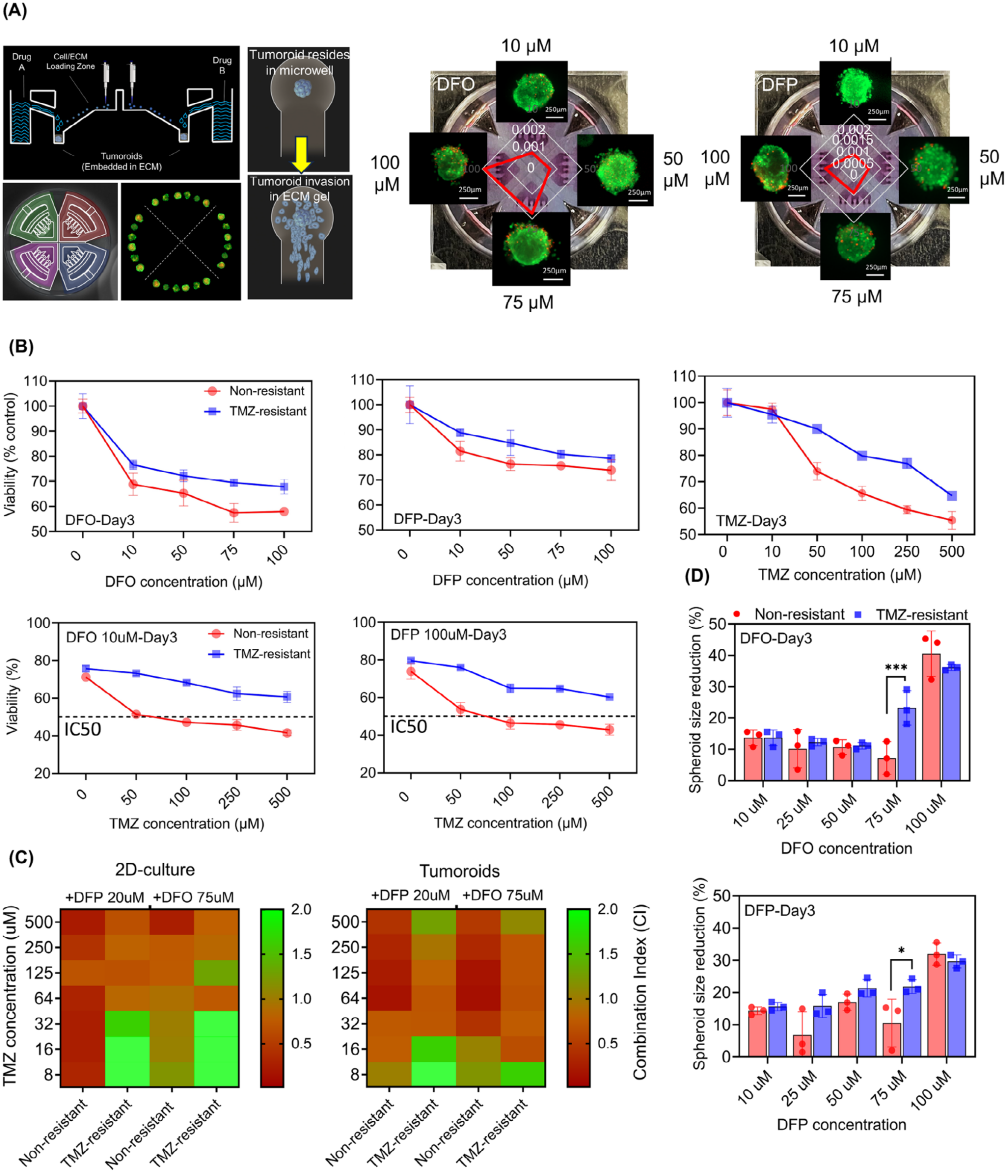
Cotreatment of TMZ‐resistant tumoroids with iron chelators and TMZ shows synergic effects. In‐vitro hGBM tumoroid in‐a‐well model was used to recapitulate the physiological relevant condition. A) Schematic side‐view of the microfluidic‐integrated culture plate (MiCP) for tumoroid array fabrication. Live‐dead (red) fluorescent imaging of tumoroids in each quadrant of the MiCP after treatment with varying concentrations of DFO and DFP along with semi‐quantified dead cells within the tumoroids. B) Change in the viability of tumoroids in response to single treatments (top panels) and combinations of either DFO or DFP with TMZ (bottom panels). C) Heat maps of CI index variation for combinations of DFO or DFP with varying concentrations of TMZ in 2D‐cultre (left) and 3D‐model (right). D) Size reduction in tumoroids in response to diffident dosages of DFO (top) and DFP (bottom). N = 3 biological independent experiments and statistically significant at *p‐value < 0.05 and *** *p*‐value < 0.001.

Using a microfluidic‐integrated culture plate with U‐shaped microcavities, we created an ECM‐incorporated tumoroid model (**Figure** **2**A‐left). These microcavities were connected to an open‐surface microchannel, allowing for the administration of specific types and concentrations of therapeutic drugs to each quadrant. Each quadrant of the culture plate contains six microwells, serving as replicates for each experiment. We studied the effects of iron chelators on viability, growth, and invasion of non‐resistant and TMZ‐resistant GBM tumoroids treated with concentrations ranging from of 10 to 100 µM of DFO and DFP. Live‐dead imaging of tumoroids in response to the various concentrations of drugs indicated an increasing population of dead cells with increasing drug concentrations in both DFP and DFO treatment conditions (Figure 2A‐right).

The reduction in tumoroid metabolic activity following single treatments with either DFO, DFP, or TMZ is depicted in Figure 2B‐top panels. DFO exhibited higher toxicity, although no half maximal inhibitory concentration (IC50) was reported for any of the single treatments. Significant correlations were observed in DFO‐treated TMZ‐resistant (*p* < 0.05 and R = 0.99), DFP‐treated non‐resistant (*p* < 0.05 and R = 0.93) and TMZ‐resistant (*p* < 0.05 and R = 0.98) tumoroids. In combination treatment of non‐resistant tumoroids with iron chelators and TMZ, the IC50 was observed for 10 µM DFO + 100 µM of TMZ (*p* < 0.05 and R = 0.83) and 100 µM DFP + 100 µM of TMZ (Figure 2B‐bottom panels, and Figure S4A, Supporting Information). No IC50 was reported for the combination treatment of TMZ‐resistant tumoroids. To evaluate the synergistic effect of combination treatments, variation of combinational inhibitory (CI) index was measured for co‐treatment of tumoroids with either 75 µM of DFO or 20 µM of DFP and a range of TMZ concentrations (Figure 2C).^[^ ^37^ ^]^ Both chelators showed synergistic effect with ranges of TMZ concentration, where a stronger effect was observed in non‐resistant cells compared to TMZ‐resistant cells. Notably, the combination treatment of TMZ‐resistant cells showed a stronger synergism in tumoroid model compared to 2D‐culture.

To assess the inhibitory effect of iron‐chelator drugs on tumoroid progression, we measured reductions in tumoroid size (Figure 2D). Significant reductions of 40% in DFO‐treated and 30% in DFP‐treated tumoroids (*p* < 0.05 and R = 0.91) were observed following the treatment with 100 µM of the drugs. Overall, higher size reductions were noted in TMZ‐resistant tumoroids compared to non‐resistant ones across most concentrations.

### 2.3 Iron Chelators Significantly Reduced the Invasiveness of Tumoroids

Activating invasion is an important hallmark of cancer.^[^ ^38^ ^]^ Tumor infiltration into surrounding tissues is a key indicator of tumor aggressiveness and metastatic potential. Monitoring invasion length allows clinicians to evaluate the efficacy of therapies in controlling tumor spread and metastasis.

**Figure 3:**
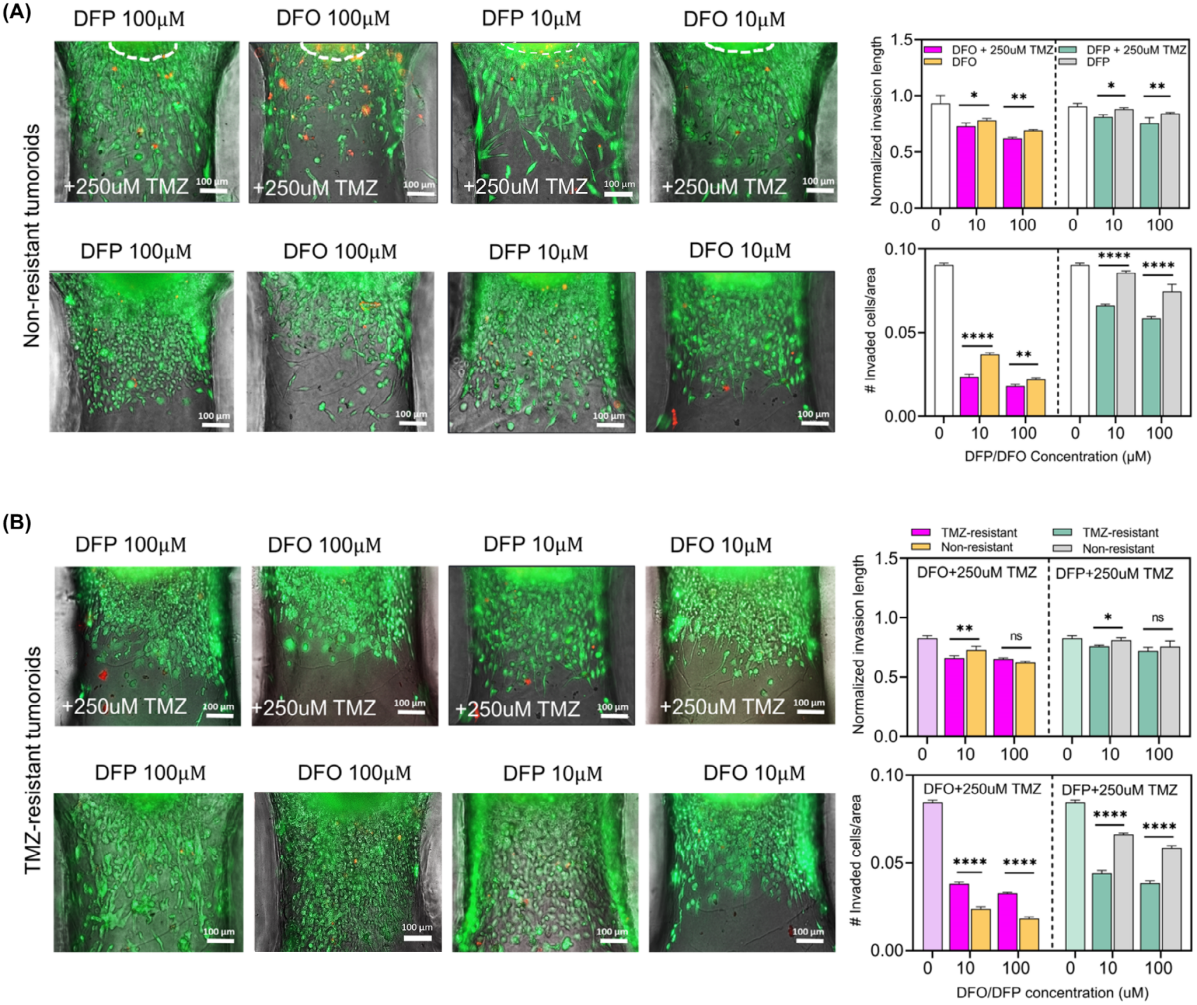
Tumoroids‐in‐a‐well model enables invasion analysis of the ECM enclosed tumor models in response to the different combination treatment conditions. A) Fluorescent images of the invaded non‐resistant tumor cells from the tumoroid with the ECM matrix in response to different combined and single treatment conditions. B) Fluorescent images of the invaded resistant tumor cells from the tumoroid with the ECM matrix in response to different combined and single treatment conditions. Invasion behavior of the tumoroids within the matrix was quantified through measurement of the relative invasion length of them compared to the primary tumoroid size and number of invaded cells per certain area.^[^ ^114^ ^]^ N = 3 biological independent experiments and statistically significant at *p‐value < 0.05, **p‐value < 0.01, ***p‐value < 0.001, and ****p‐value < 0.0001.

Here, we studied the effect of iron chelators on the invasion of GBM tumoroids embedded in the ECM hydrogel. We quantified the extent of invasion and the number of cells per area invaded into the ECM in both non‐resistant and TMZ‐resistant tumoroids in response to combination treatments with 100 µM TMZ + 10 µM or 100 µM of either DFO or DFP (**Figure** **3**). These results indicated that the combined therapy significantly reduced both invasion length (top panels) and invasion density (bottom panels) of the tumoroids compared to single iron‐chelator therapy with DFO and DFP.

Additionally, the invasion length of TMZ‐resistant tumoroids treated with 100 µM TMZ + 10 µM of either DFO or DFP was significantly lower than the same treatment on non‐resistant tumoroids (Figure 3B‐top panels). Similarly, significant differences were observed in the number of invaded cells under cotreatment of tumoroids with 100 µM TMZ + 10 µM or 100 µM DFP (Figure 3B‐bottom panels). The reduction of invaded cell number in TMZ‐resistant tumoroids due to the combination of TMZ and DFO was not significantly lower compared to non‐resistant tumoroids. Overall, the combination of DFP and TMZ treatment demonstrated a significant reduction in the length and the density of the invasive cells, a promising outcome toward inhibiting the diffusion of GBM through brain tissue.

### 2.4 Iron Chelators Significantly Reduced the Viability of Patient‐Derived Recurrent GBM

**Figure 4:**
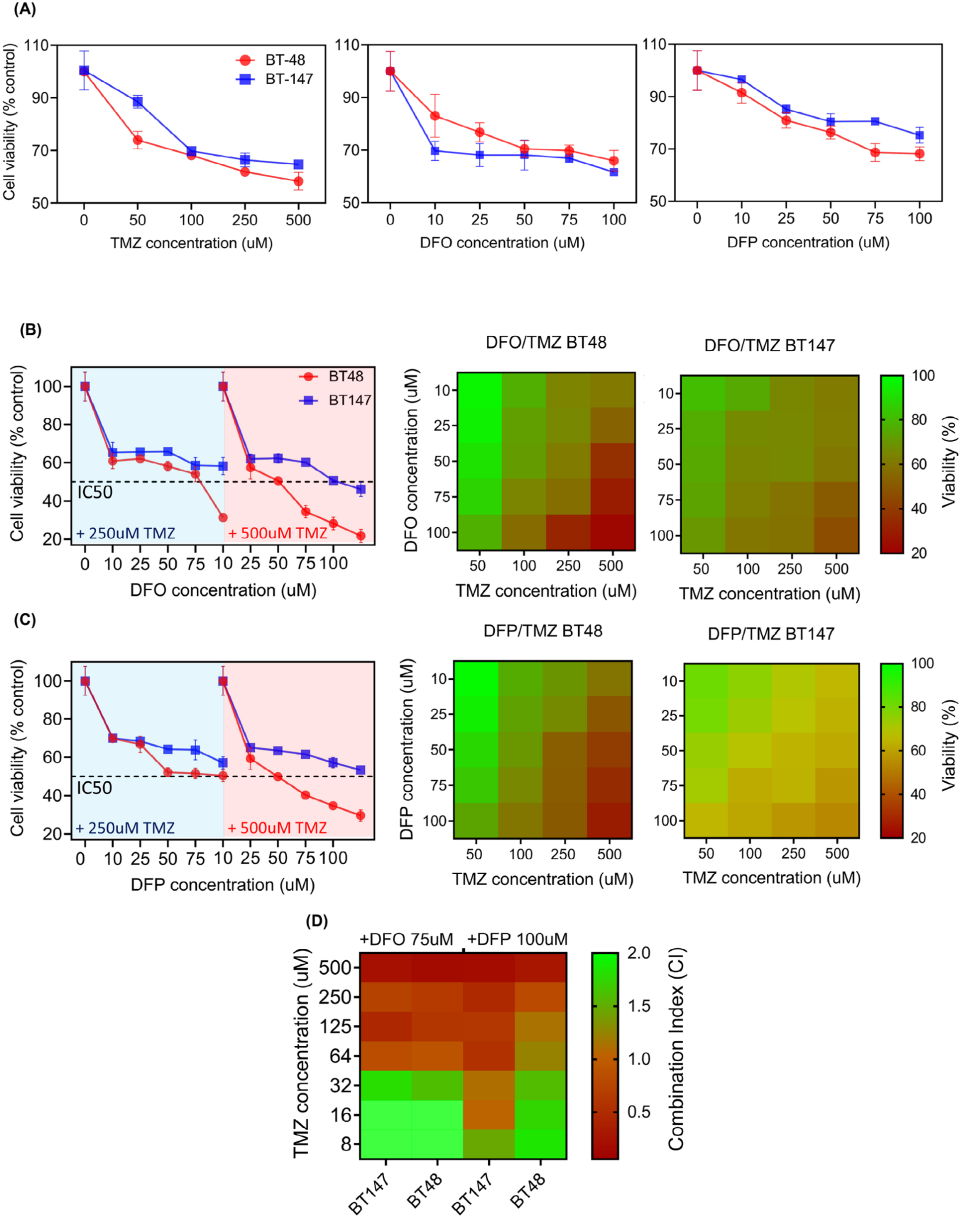
Co‐treatment of glioblastoma patient‐derived tumoroid with TMZ and iron chelators induced synergic effect. A) Change in viability in response to single treatment of (left to right) TMZ, DFO, and DFP. B) The IC50 values were associated to the combination of 250 mM TMZ + 100 mM DFO and 500 mM TMZ + 50,75100 mM DFO in non‐resistant tumoroids, and only 500 mM TMZ + 100 mM DFO in recurrent tumoroids. C) Additionally, the IC50 values were obtained for the combination of 500 mM TMZ + 25, 50, 75, and 100 mM DFP in non‐resistant tumoroids. D) Heat maps of CI index variation for combinations of DFO or DFP with varying concentrations of TMZ in patient‐derived tumoroids. N = 3 biological independent experiments.

The viability of two types of patient‐derived cells in response to single and co‐treatment with TMZ, DFO, and DFP was measured in tumoroid culture. The first cell type was derived from newly diagnosed tumors and labeled by BT‐48 (male, 68 yrs.), was compared with recurrent cells treated by radiotherapy + concurrent adjuvant TMZ chemotherapy, labeled by BT‐147 (male, 55 yrs.). Results indicated that TMZ‐treated recurrent tumoroids exhibited less sensitivity to TMZ but increased sensitivity to DFO and DFP compared to the newly diagnosed tumoroid (**Figure** **4**A). Significant correlations were observed for single treatments with DFO (BT‐48: *p* < 0.05 and R = 0.74) and DFP (BT‐48: *p* < 0.05 and R = 0.88, BT‐147: *p* < 0.05 and R = 0.80).

While no IC50 value was observed for either of the drugs in the single treatment regime, several IC50 values were associated with combination therapies. Specifically, the IC50 values for co‐treatment of the newly diagnosed tumoroid were 250 µM TMZ + 100 µM DFO, and 500 µM TMZ + 50, 75, 100 µM DFO, while for recurrent tumoroid, it was 500 µM TMZ + 100 µM DFO (Figures 4B and S4B, Supporting Information). Significant correlations were found in 250 and 500 µM DFO treatment on both newly diagnosed and recurrent tumoroids (BT‐48: *p* < 0.05, R = 0.96 and R = 0.75 respectively, BT‐147: *p* < 0.05, R = 0.77 and R = 0.90, respectively). Additionally, IC50 values were obtained for the combination of 500 µM TMZ + 25, 50, 75, and 100 µM DFP in recurrent tumoroid (Figures 4C and S4B, Supporting Information). Similar to DFO, significant correlations were found in 250 µM and 500 µM DFO treatment on both newly diagnosed and recurrent tumoroids (BT‐48: *p* < 0.05, R = 0.82 and R = 0.95, respectively, BT‐147: *p* < 0.05, R = 0.93 and R = 0.98, respectively). To evaluate the synergistic effect of combination treatments, variation of CI index was measured for co‐treatment of tumoroids with either 75 µM of DFO or 100 µM of DFP and a range of TMZ concentrations (Figure 4D). Both chelators showed synergistic effect with ranges of TMZ concentration higher than 64 µM. These findings demonstrate that the proposed combination therapy is not only effective for newly diagnosed GBM tumoroids but also improves efficacy in recurrent tumoroids.

### 2.5 TMZ‐Resistant Cells Acquired Distinctive Traits in their Response to Iron Chelators

**Figure 5:**
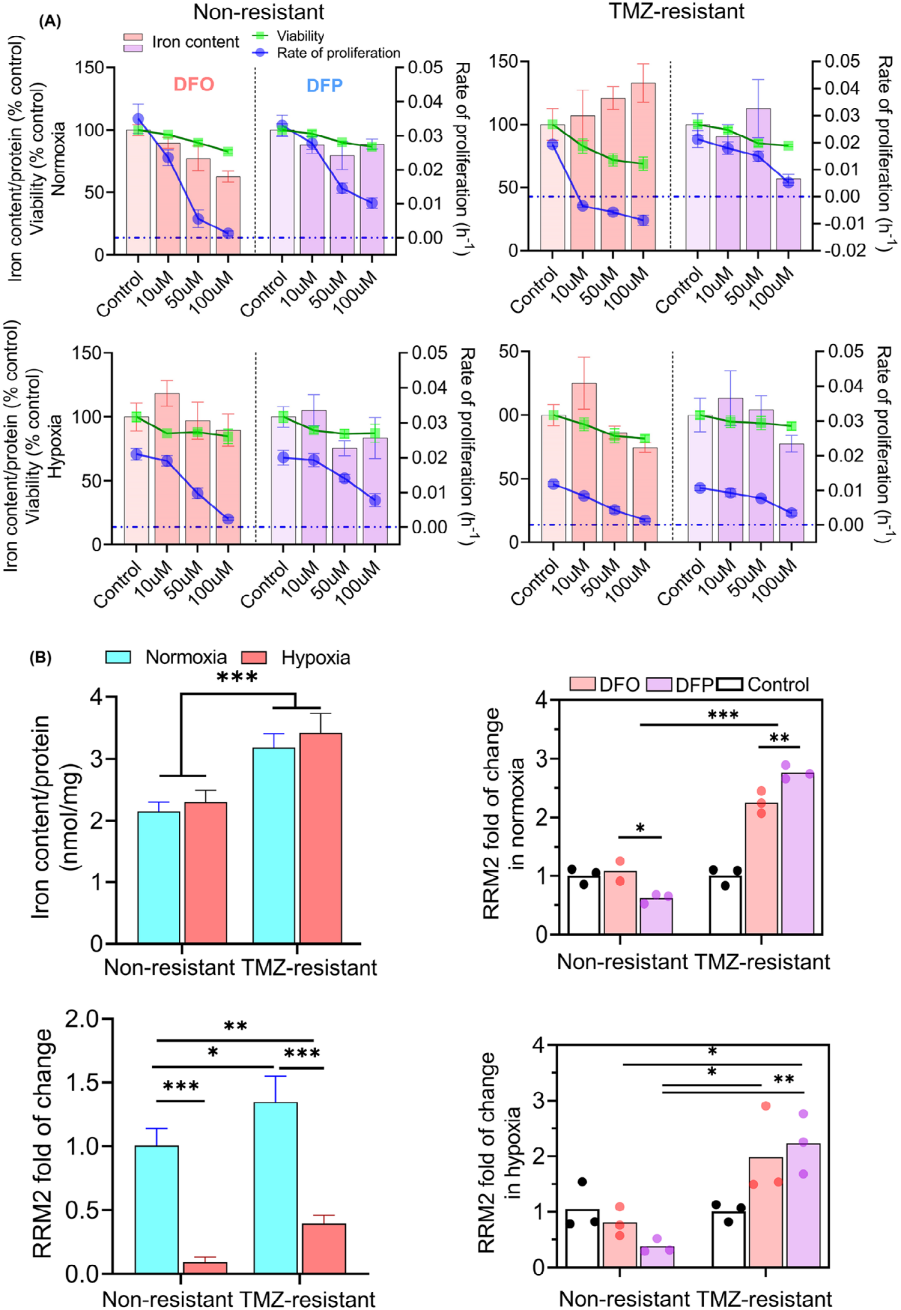
TMZ‐resistant cells exhibited different pattern of variations of in viability, proliferation rate, and ribonucleotide reductase mRNA (RRM2) expression. A) Both chelators decreased viability and proliferation, with DFO showing greater efficacy, particularly in normoxic TMZ‐resistant cells. DFO reduced intracellular iron in normoxic non‐resistant cells but increased intracellular iron in TMZ‐resistant cells. Hypoxic cells showed increased resistance to chelators (A‐bottom panels). Notably, TMZ‐resistant cells exhibited higher intracellular iron (B‐top left). Furthermore, TMZ‐resistant cells upregulated RRM2 expression in both normoxia and hypoxia (B‐bottom left). Similar trend was observed in TMZ‐resistant cells in response to DFO and DFP in both normoxia and hypoxia (B‐right panels). Both chelators changed RRM2 expression, with DFP showing a stronger effect. In non‐resistant cells, RRM2 expression decreased with chelator treatment, more significantly with DFO. N = 3 biological independent experiments and statistically significant at *p‐value < 0.05, **p‐value < 0.01, ***p‐value < 0.001, and ****p‐value < 0.0001.

We studied the effects of iron deficiency in 2D culture for preliminary dosage screening of iron chelators. Since tumors often develop hypoxic zones due to limitation in oxygen diffusion, we examined both normoxic and hypoxic states of cells. Treatment with increasing doses of the iron chelators DFO and DFP reduced cell viability and proliferation rates of both non‐resistant and TMZ‐resistant cells under hypoxic and normoxic conditions (**Figure** **5**A). However, TMZ‐resistant cells were more sensitive to treatment. Compared to cells in normoxic conditions, cells exposed to hypoxic conditions maintained higher viability (Figure 5A, bottom) even after 72 h treatment with iron chelators (Figure S6A, Supporting Information). Overall, DFO treatment was more effective in reducing proliferation rates compared to DFP, and the highest reductions in viability and proliferation rate were observed in DFO‐treated normoxic TMZ‐resistant cells (Figure 5A‐top right). Of note, both cell types displayed greater susceptibilities to DFO than to DFP, and the application of DFO to TMZ‐resistant cells resulted in pronounced negative proliferation rates (Figure 5A‐top right). Significant correlations were found in the viability of DFO‐treated (*p* < 0.05 and R = 0.98) and DFP‐treated (*p* < 0.05 and R = 0.94) normoxic non‐resistant cells, as in the proliferation rate of DFO‐treated (non‐resistant: *p* < 0.05 and R = 0.98, TMZ‐resistant: *p* < 0.05 and R = 0.93) and DFP‐treated (non‐resistant: *p* < 0.05 and R = 0.99, TMZ‐resistant: *p* < 0.05 and R = 0.96) cells, as well as DFP‐treated normoxic TMZ‐resistant cells (*p* < 0.05 and R = 0.95). Other conditions did not exhibit a significant correlation in viability and proliferation rate.

To investigate the effects of chelator treatment further, intracellular iron content was evaluated. Both cell lines cultured in normoxic conditions exhibited a reduction in intracellular iron when treated with iron chelators in most cases (Figure 5A‐top panels). A dose‐dependent reduction was observed with DFO treatment of non‐resistant cells (*p* < 0.05 and R = 0.96), and the most effective condition was 100 µM, reducing iron levels by 38% (Figure 5A‐top left). Cells cultured in hypoxic conditions also showed a reduction in intracellular iron in response to DFO and DFP (Figure 5A‐bottom panels), and a significant correlation observed in DFO‐treated non‐resistant cells (*p* < 0.05 and R = 0.97).

One notable exception to these trends was DFO‐treated TMZ‐resistant cells, the same conditions that produced the greatest effect on cell viability. Under these conditions, intracellular iron was increased by 30% (Figure 5A‐top right). A comparison between TMZ‐resistant and non‐resistant cells revealed relatively higher intracellular iron content in TMZ‐resistant cells overall (Figure 5B‐top left). Intracellular levels of zinc, manganese, magnesium, and copper were not significantly changed in response to treatment with chelators in any condition tested (Figure S7, Supporting Information). These findings emphasize the heightened responsiveness of normoxic TMZ‐resistant cells to DFO treatment.

Expanding on the distinct responses observed between TMZ‐resistant and non‐resistant cells, we measured the gene expression of RRM2, the rate‐limiting enzyme in the synthesis of deoxyribonucleoside triphosphates (dNTPs) crucial for DNA synthesis and repair.^[^ ^39^, ^40^ ^]^ Since iron is an essential cofactor for RRM2 activity, intracellular iron changes may have an impact on RRM2 levels and downstream proliferation.^[^ ^41^ ^]^ TMZ‐resistant cells upregulated RRM2 expression in both normoxia and hypoxia (Figure 5B‐bottom left). The same result was observed following treatment with DFO and DFP (Figure 5B‐right panels). Unlike TMZ‐resistant cells, non‐resistant cells downregulated RRM2 expression in response to chelator treatment, with DFO demonstrating higher efficacy.

### 2.6 TMZ‐Resistant Cells Exhibited Lower HIF1‐α and Higher ROS Accumulations Compared to Non‐Resistant Cells in Response to Iron Chelators

Given the pronounced impact of hypoxia on cellular responses to iron deficiency, we further elucidated the role of hypoxia by measuring glucose uptake, a marker of cellular metabolism, and ROS production in both non‐resistant and TMZ‐resistant cells under hypoxic conditions.

**Figure 6:**
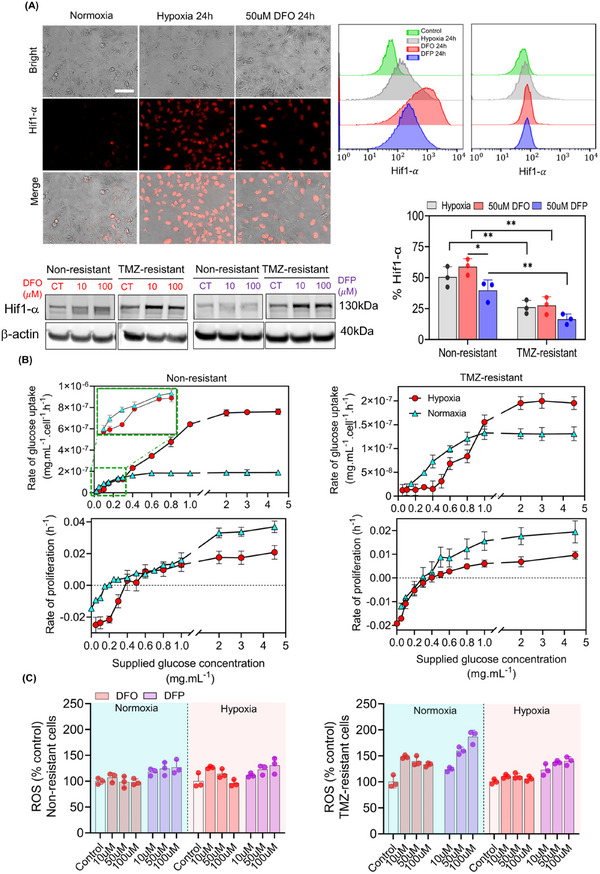
DFO and DFP upregulate Hif1‐α expression and ROS generation in non‐resistant and TMZ‐resistant cells. A) Hif1‐α expression, imaged by IF microscopy (A‐left panels) and flow cytometry (A‐right panels) as well as western blotting (the protein, for the same experimental conditions across the lanes, came from the same lysate master mix, used in this figure and in Figures 7 and 8), revealed upregulation in response to 24 h of hypoxia and 50 µM DFO and DFP treatments. Flow cytometry quantification of Hif‐1α accumulation, indicating higher levels in DFO‐treated non‐resistant cells compared to DFP and hypoxia, with a similar trend observed in TMZ‐resistant cells. Glucose uptake analyses (B‐top panels) highlighted a pronounced difference between non‐resistant and TMZ‐resistant cells, with varying glucose concentrations affecting proliferation rates (B‐bottom panels), suggesting a critical threshold and different responses under hypoxic and normoxic conditions. This denotes the high sensitivity of normoxic TMZ‐resistant cell to DFO. In contrast, hypoxic TMZ‐resistant cells were less sensitive to all tested concentrations of DFO and DFP. C) Non‐monotonic dose‐dependent increase of ROS in DFO‐treated non‐resistant and TMZ‐resistant cells. DFP induced increases in ROS across all conditions. Hypoxia reduced the effect of both DFO and DFP in TMZ‐resistant cells. N = 3 biological independent experiments and statistically significant at *p‐value < 0.05 and **p‐value < 0.01. Scale bar is 100 µm.

Expression of HIF‐1α was imaged by immunofluorescence (IF) microscopy, flow cytometry analysis, and western‐blotting (**Figure** **6**A). HIF1‐α accumulation was shown in non‐resistant cells exposed to either 24 h of hypoxia, or normoxia with 50 µM DFO (Figure 6A‐left panels), or DFP (Figure S5A, Supporting Information). Similarly, HIF1‐α accumulated in response to DFO or DFP treatment under hypoxic conditions (Figure S5A, Supporting Information). When treated with DFO or DFP, both non‐resistant and TMZ‐resistant cells upregulated the expression of HIF1‐α in a similar manner to cells exposed to hypoxic conditions. However, combined treatment with either DFO or DFP in hypoxia did not result in a significant change in HIF1‐α expression. Additionally, the expression of HIF1‐α was quantified by flow cytometry analysis (Figure 6A‐right panels, Figure S5B, Supporting Information). 24 h of incubation in hypoxia and treatment with 50 µM DFO and DFP induced accumulation of HIF1‐α in non‐resistant cells by 50%, 59%, and 44%, and in TMZ‐resistant cells by 26%, 37%, and 21%, respectively. Cells treated with DFO exhibited a higher expression level of HIF1‐α compared to both DFP treatment and hypoxia incubation in non‐resistant cells. A similar trend was observed in TMZ‐resistant cells. Across all treatments, non‐resistant cells showed a greater accumulation of HIF1‐α compared to TMZ‐resistant cells. These results indicate that both oxygen deprivation and intracellular iron depletion can stimulate the expression of HIF1‐α.

To understand the response of non‐resistant versus TMZ‐resistant cells to nutrient perturbation, we quantified glucose uptake and proliferation rates of these cells under hypoxic and normoxic conditions. The glucose supply was in the range of 0 to 4.5 mg mL^−1^, clinically equivalent to 450 mg dL^−1^. While 450 mg dL^−1^ exceeds the normal blood glucose range of 70–144 mg dL^−1^, mean glucose levels it in GBM patients can reach as high as 459 mg dL^−1^.^[^ ^42^ ^]^

In all conditions, the glucose uptake and the proliferation rate were increased with glucose concentration and plateaued at higher concentrations (Figure 6B). This increase was more pronounced in non‐resistant cells compared to TMZ‐resistant cells (Figure 3B‐top panels). The critical threshold of transitioning from negative to positive proliferation rates was higher for hypoxic cells (0.5 mg mL^−1^) compared to normoxic cells (0.2 mg mL^−1^) (Figure 6B‐bottom panels). However, the overall proliferation rate was higher for the cells grown under normoxic conditions. Similar trends were observed for TMZ‐resistant cells with less variations in concentration‐dependent uptake and proliferation rates.

An inverse dose‐dependent correlation in ROS level was observed in DFO‐treated non‐resistant and TMZ‐resistant cells (Figure 6C). However, DFP induced increases in ROS across all conditions. Hypoxia reduced the effect of both DFO and DFP in TMZ‐resistant cells. A significant correlation was observed in the dose of DFP versus ROS level in hypoxic non‐resistant (*p* < 0.05 and R = 0.91) and normoxic TMZ‐resistant (*p* < 0.05 and R = 0.94) cells. The higher concentration of chelators, the more ROS generation was observed after 72 h of treatment in all tested conditions in both DFO and DFP treatments (Figure S6B, Supporting Information).

### 2.7 Iron Chelators Induced Autophagy in Normoxia but Inhibited Autophagic Flux in Hypoxia

**Figure 7:**
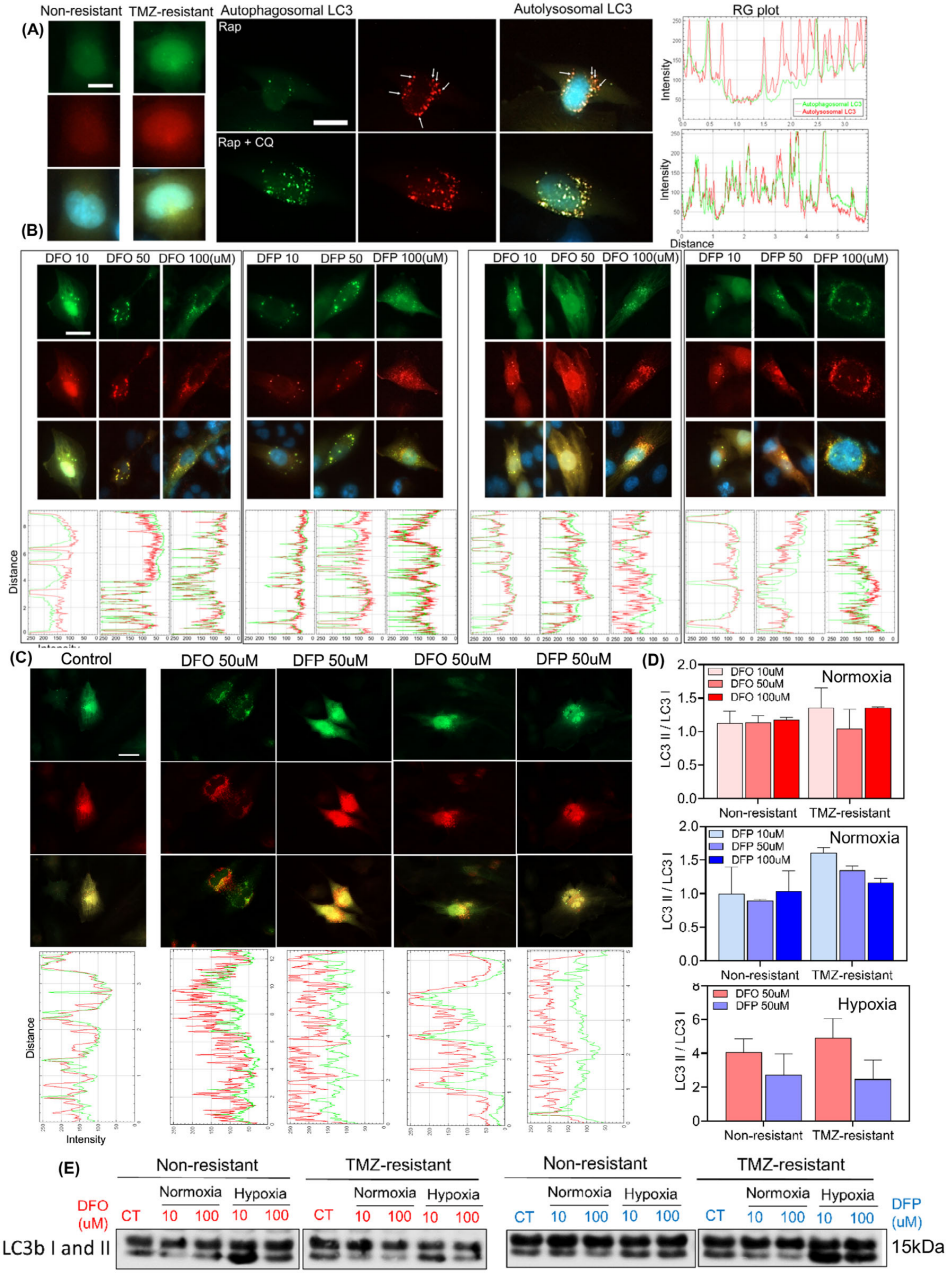
DFO and DFP induce autophagy but inhibit autophagic flux in normoxia, whereas they induce autophagy and regulate the autophagic flux in hypoxia. A) Autophagic flux visualization using GFP‐RFP‐LC3 adenovirus in U251 NR and TMZ‐resistant cells. Positive control cells treated with 750 nM Rap and 750 nM Rap + 30 µM CQ. The autophagic flux was observed by imaging autolysosomes (red puncta) in cells treated with Rap, while autophagy inhibition was monitored by localized mRFP/GFP (yellow puncta) in Rap + CQ treated cells. B‐left) Effects of 10, 50, and 100 µM of DFO and DFP treatments on NR cells showed co‐localization of mRFP‐GFP (yellow puncta) in all concentrations of DFO and DFP, which indicates the inhibition of autophagy flux. B‐right) The same treatments were performed on TMZ‐resistant cells. Similar to NR cells, autophagy inhibition by DFO and DFP was observed in TMZ‐resistant cells. C) Different variation in autophagic flux was observed in hypoxic non‐resistant and TMZ‐resistant cells. Iron chelators induced autophagy, but regulated the flux, unlike normoxic cells. D) The ratio of LC3II to LC3I quantified by counting red and yellow puncta confirmed the inhibition and regulation of autophagic flux in normoxia and hypoxia, respectively. E) These results were also confirmed by WB. (Note that β‐actin was probed separately and each of the experimental conditions across the lanes came from the same lysate master mix; see further explanation about β‐actin in Immunoblotting section of Experimental Methods). N = 3 biological independent experiments. Scale bars are 20 µm.

Hypoxic GBM cells are more resistant to traditional therapy which necessitates further investigation of their mechanisms of resistance during therapy, such as upregulation of autophagic pathways. Green fluorescent protein‐red fluorescent protein‐microtubule associated protein 1 light chain 3 (GFP‐RFP‐LC3) adenovirus was used to monitor autophagic flux as a measure of autophagic degradation. Instability of GFP fluorescence in autolysosome acidic environments, where mRFP is relatively stable, allowed for the characterization of the autophagic compartments. Lysosomes fusion was visualized by tracking the loss of GFP fluorescence. The number of autophagosomes was counted by co‐localized mRFP/GFP signals (yellow puncta), while autolysosomes were identified by red puncta. Time‐matched control cells showed double positive RFP/GFP‐LC3 signals in the cytoplasm (**Figure** **7**A).

A concentration‐dependent co‐localization of mRFP‐GFP (yellow puncta) was observed in normoxic DFO‐ and DFP‐ treated non‐resistant cells (Figure 7B‐left), indicating the inhibition of autophagic flux, confirmed by intensity plots (Figure S8A, Supporting Information). Similar to non‐resistant cells, autophagic flux was inhibited by DFO and DFP in normoxic TMZ‐resistant cells (Figure 7B‐right). Interestingly, analyses of autophagic response under hypoxic conditions revealed that neither of the iron chelators inhibited the autophagic flux in non‐resistant and TMZ‐resistant cells (Figure 7C).

The ratio of LC3‐II to LC3‐I, equivalent to the ratio of red to yellow puncta, is a valuable indicator of autophagic activity. Monitoring this ratio provides insights into autophagic flux (Figure 7D). Higher ratios in hypoxic cells indicated lysosome fusion, indicative of autophagic flux regulation. These results were also confirmed by WB (Figures 7E and S8B, Supporting Information). Overall, these findings show that iron chelators induce autophagy but block the autophagic flux in normoxic cells, while hypoxic cells are able to maintain autophagic flux.

### 2.8 TMZ‐Resistant and Non‐Resistant Cells Exhibit Blebbing in Response to Iron Chelators

**Figure 8:**
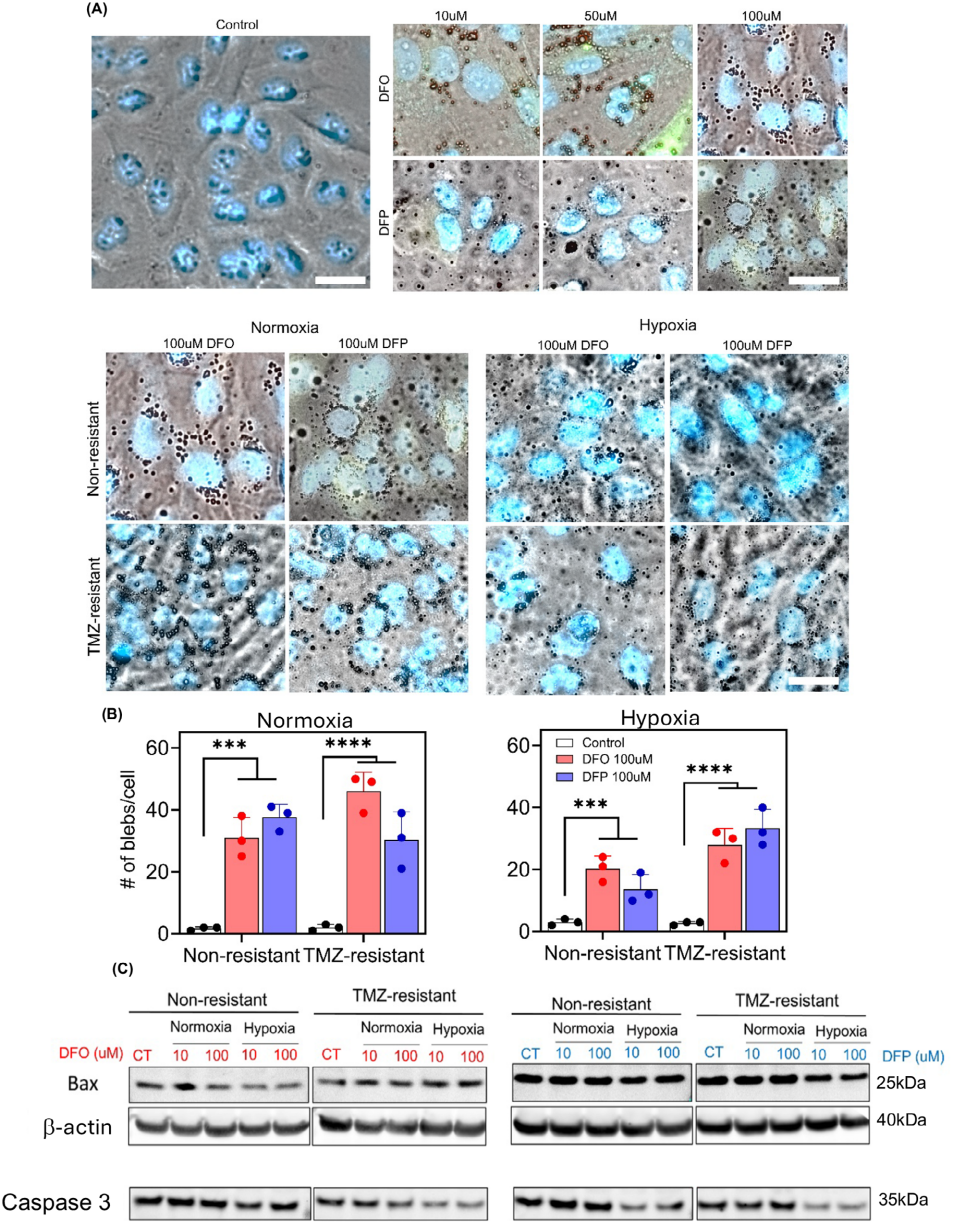
Higher expression of bax in DFO‐treated TMZ‐resistant cells, while hypoxia reduces the expression of caspase3. A) Dose dependent induction of cell blebbing was observed in normoxic non‐resistant cells. B) Hypoxia reduced cell blebbing in both non‐resistant and TMZ‐resistant cells. C) Expression of bax and caspase3 in normoxic and hypoxic non‐resistant and TMZ‐resistant cells in response to 10 and 100 mM of DFO and DFP. (Note that β‐actin was probed separately for Caspase 3 and each of the experimental conditions across the lanes came from the same lysate master mix; see further explanation about β‐actin in Immunoblotting section of Experimental Section). Bax expression was increased only in response DFO in normoxic and hypoxic TMZ‐resistant cells. However, bax was not increased in non‐resistant cells under any treatment. Although neither of the chelators showed significant increase in the expression of caspase3, it was reduced in hypoxia. N = 3 biological independent experiments and statistically significant at ***p‐value < 0.001 and ****p‐value < 0.0001. Scale bars are 20 µm.

Cell blebbing was observed in both DFO and DFP‐treated non‐resistant and TMZ‐resistant cells. Higher concentrations of the drugs induced a larger number of blebs (**Figure** **8**A). Both non‐resistant and TMZ‐resistant cells underwent blebbing with hypoxia counteracting (Figure 8B). The expression of bax and caspase3 was investigated using WB (Figure 8C), and further analyzed by densitometry (Figure S6C, Supporting Information). Bax expression was increased in DFO‐treated normoxic and hypoxic TMZR cells. However, the treatment did not increase bax expression in non‐resistant cells. Overall, DFP treatment did not affect bax expression in either cell type. Neither of the chelators showed significant increase in the expression of caspase3.

## 3 Discussion

Our results highlight strong metabolic differences between TMZ‐resistant and non‐resistant GBM cells, whether cultured in 2D or 3D. Treatment of these cells with the clinical iron chelators DFO and DFP in most conditions causes these cells to become iron deficient, lose viability and die, which could be exploited to sensitize resistant GBM to chemotherapy. We observed significant variation between TMZ‐resistant and non‐resistant GBM cells in intracellular iron, viability, proliferation, HIF1‐α expression, ROS level, and autophagy (Figure S9, Supporting Information). The results did not confirm any particular type of cell death. The CI index values obtained for the co‐treatment of non‐resistant and TMZ‐resistant tumoroids with ranges of TMZ and DFO or DFP showed synergistic effects. The combined therapy also induced a significant reduction in the size and the relative invasion length of the tumoroids. These results suggest that depriving iron and oxygen may lead to different variations in metabolic pathways in TMZ‐resistant versus non‐resistant cells.

TMZ‐resistant cells revealed different proteomic and secretome metabolic profiles compared to non‐resistant cells derived from either 2D or 3D cells. Acquiring TMZ resistance is linked to reduced expression of ribosomal and lysosomal proteins, along with increased expression of genes related to vesicular transport and oxidative phosphorylation (OXPHOS), emphasizing the importance of iron and oxygen in cancer cells experiencing normoxic conditions.^[^ ^43^, ^44^ ^]^ This is consistent with previous studies showing that resistant glioma stem cells depend on OXPHOS for survival and that targeting this pathway could be effective in overcoming resistance.^[^ ^45^, ^46^, ^47^ ^]^ Additionally, the lower expression of lysosomal and ribosomal proteins suggests alterations in cellular recycling mechanisms and protein synthesis, which have been previously shown to aid in acquiring drug resistance.^[^ ^48^, ^49^ ^]^ We observed higher expression of aconitase (IRP1), a pivotal enzyme involved in the regulation of TCA cycle, lipogenesis, oxidative phosphorylation, and ATP production. This underscores the significant influence of intracellular iron levels on the adapted metabolism observed in TMZ‐resistant tumoroids. Collectively, these changes showed that TMZ‐resistant cells have altered cellular metabolism, trafficking, and cytoskeletal components that may enable them to become resistant to TMZ.

3D‐cultured resistant cells also showed distinct metabolic shifts in the 3D TME, similar to their monolayer counterparts. It is well known that in hypoxic conditions, glucose utilization via the Warburg effect, and iron metabolism all play a critical role in tumor formation and metastasization of cancers,^[^ ^32^ ^]^ underscoring the critical role of metabolic adaptations in both acquired drug resistance and tumor formation. Invasion pattern is another major difference between non‐resistant and TMZ‐resistant GBM tumors associated with the resistance. We observed that non‐resistant tumoroids formed a star‐shaped invasion pattern with elongated protrusions into the healthy areas of the matrix, similar to diffuse gliomas. Conversely, TMZ‐resistant tumoroids showed scattered growth with dispersed protrusions. A closer examination of the invading cells revealed that the elongated non‐resistant cells feature clustered actin and finger‐like projections, along with filopodia protrusions indicative of a mesenchymal phenotype.^[^ ^50^ ^]^ In contrast, resistant cells were rounded with shallow, actin‐rich bleb‐like projections and podosome protrusions, characteristic of an amoeboid phenotype.^[^ ^51^, ^52^ ^]^ Conversely, resistant cells were rounded with bleb‐like projections that are shallow and actin‐rich, podosome protrusions, as seen in cells with amoeboid phenotype.^[^ ^51^, ^52^ ^]^ Additionally, the migration plasticity in TMZ‐resistant cells confirmed decreased adhesion properties related to mesenchymal to amoeboid transition (MAT), a characteristic of stem cells.^[^ ^53^, ^54^, ^55^ ^]^ In TMZ‐resistant tumoroids, the levels of cytokines integral to the immune system such as TGF‐alpha, IP10, and IL21 were lower compared to non‐resistant tumoroids. The decreased levels of these cytokines may contribute to the aggressiveness of tumors.

Our findings also indicate that TMZ‐resistant tumoroids displayed elevated proteins of mitochondrial oxidation and cell viability, along with decreased secretion of pro‐inflammatory cytokines and reduced attachment properties. OXPHOS provides a higher energy level for cells, which likely explains why 3D culture is associated with drug resistance in tumoroids due to increased oxidative drug extrusion.^[^ ^56^ ^]^ A recent proteomic analysis comparing drug‐resistant melanoma monolayers‐ and 3D‐cultures also found higher OXPHOS in 3D‐culture condition.^[^ ^57^, ^58^ ^]^ Similar findings have been observed in colorectal cancer and pancreatic ductal adenocarcinoma. The variabilities that we observed between chemo‐resistant and non‐resistant cells in normoxia/hypoxia may possibly shape the differences seen in heterogeneous GBM tumor.

Additionally, the stronger inhibitory effect of TMZ combined with iron chelators (DFO or DFP) on the invasive ability of TMZ‐resistant GBM cells compared to TMZ‐sensitive cells can be attributed to the heightened dependence of resistant cells on iron. Iron chelation interrupts critical processes such as DNA repair, where iron is a necessary cofactor for enzymes like MGMT, exacerbating the stress induced by TMZ and further impairing cellular repair pathways. Iron plays a pivotal role in promoting GBM cell invasion by regulating key signaling pathways such as MAPK/ERK and PI3K/AKT, which drive cell migration.^[^ ^59^ ^]^ Elevated iron levels support the activity of HIF‐1α, which enhances extracellular matrix remodeling,^[^ ^60^ ^]^ angiogenesis, and motility, as well as metalloproteinases (MMPs) that degrade the extracellular matrix. TMZ‐resistant GBM cells, with their higher baseline oxidative stress, are particularly vulnerable to the additional oxidative damage caused by iron chelation, further reducing their invasive potential.

TMZ‐resistant cells showed relatively higher intracellular iron content compared to non‐resistant cells, which might help them to enhance OXPHOS and enable their stem‐cell like characteristics.^[^ ^61^, ^62^ ^]^ They also upregulated RRM2 transcription in response to iron chelators, while non‐resistant cells are unable to compensate by upregulating RRM2 transcription to the same extent. In both cell types, DFP showed stronger effects that can be explained by its lipophilicity. These findings suggest that while TMZ‐resistant cells are more sensitive to iron chelators, they possess an enhanced capacity to respond at the gene expression level by upregulating enzymes involved in DNA synthesis. A possible mechanism for this increase may be a feedback loop induced by changes in cellular iron content or direct effect of chelators on enzyme activity, supported by the increment of RRM2 expression in TMZ‐resistant cells. This could explain the higher sensitivity of TMZ‐resistant cells to iron chelator treatment compared to non‐resistant cells.

Treatment with both iron chelators changed the metabolism of GBM cells. For example, chelators reduced cellular viability and proliferation in TMZ‐resistant cells and non‐resistant cells but were most effective in normoxic conditions. Treatment with DFO was particularly effective in reducing the viability of TMZ‐resistant cells, but the mechanism may be dependent on the presence of oxygen. In normoxic conditions, these cells exhibited increased intracellular iron, along with RRM2 expression and ROS, but lower levels of HIF‐1α compared to non‐resistant cells, indicating that they are not iron limited and may instead be experiencing toxic side effects of high iron, or the direct inhibition of mitochondrial electron transport by chelators, evidenced by increased ROS.^[^ ^63^ ^]^ Supporting this, these cells were not promoting autophagic flux as much as their hypoxic counterpart, but instead displayed increased blebbing of cell death. The increase in intracellular iron level could be due to reduced iron efflux transport by downregulation of proteins responsible for iron export, such as ferroportin.^[^ ^64^, ^65^, ^66^ ^]^ The pro‐apoptotic proteins Bax and caspase3 were not increased. These findings suggest that the method of cell death observed here may not primarily occur through mitochondrial pathways in these cells and could instead be occurring through other regulated pathways such as ferroptosis or autophagy‐dependent cell death (ADCD), highlighting the complex crosstalk between autophagy and cell death.^[^ ^67^, ^68^, ^69^ ^]^ It could be that iron chelators induce a limited form of autophagy as a potential survival mechanism that is doomed to failure from lack of resources inducing ADCD. ADCD has been reported for another iron chelator, VLX600, by prominent induction of bulk autophagy and mitophagy in GBM.^[^ ^70^ ^]^ Treatment with iron chelators in hypoxic conditions reduced intracellular iron, but RRM2 expression and autophagic flux were increased, suggesting these cells were experiencing iron deficiency. Interestingly, although cell blebbing was lower in hypoxia, bax was also upregulated. Iron chelation has been shown in other cancer models to downregulate the anti‐apoptotic Bcl2 protein, MCL‐1, which could be a part of the mechanism making TMZ‐resistant cells more vulnerable to death and be an important part of the mechanism of cell death in iron deficient GBM cells.^[^ ^71^, ^72^, ^73^ ^]^ Additionally, as both oxygen deprivation and intracellular iron depletion can stimulate the expression of HIF1‐α, the latter sometimes called “chemical hypoxia,” it is important to investigate the downstream signaling pathways and transcriptional changes mediated by HIF1‐α in response to DFO and DFP treatment. Our data indicate that iron chelators modulate HIF‐1α expression in TMZ‐resistant GBM cells, showing reduced levels compared to non‐resistant cells rather than a complete suppression of HIF‐1α. These findings suggest that while TMZ‐resistant cells can still accumulate HIF‐1α under iron chelation, the reduced expression may reflect the interplay between altered metabolic states and impaired iron availability. We also observed that DFO and DFP demonstrated high selectivity in removing iron, increasing ROS and inducing cell death without affecting homeostatic levels of other metals like copper, magnesium, manganese, and zinc, making them promising candidates for cancer therapeutics.

The interplay between iron metabolism and ROS is an important aspect of using iron chelators in cancer therapy. Iron excess or deficiency can result in the generation of ROS.^[^ ^24^ ^]^ While high levels of iron can promote cancer cell growth and help them to meet their need of essential factors for proliferation, they may contribute to Fenton chemistry yielding hydroxy radicals and causing ferroptosis. On the other hand, chelator‐induced iron deficiency can lead to mitochondrial dysfunction by decreasing mitochondrial iron or inhibiting electron transport, as described above, leading to superoxide radical leakage from the electron transport chain (ETC) and causing considerable damage and cell death. However, cancer cells can employ various mechanisms to counteract ferroptosis via upregulation of ferroptosis‐suppressing pathways.^[^ ^74^ ^]^ This dual role of iron as both an anti‐ and pro‐survival element necessitates a high level of investigation in developing 3D models of GBM to understand therapeutic strategies interfering with iron metabolism.

Overall, combination therapy with iron chelators and TMZ holds promise in disrupting pathways involved in TMZ resistance and altering the invasiveness of these cells. DFP's lipophilic nature allows it to penetrate cell membranes and the BBB.^[^ ^75^ ^]^ DFO, widely known for its efficacy in iron binding and ability to decrease non‐transferrin‐bound iron^[^ ^76^, ^77^ ^]^ that could penetrate the BBB,^[^ ^78^, ^79^ ^]^ is not readily BBB permeable but can cross the BBB after systemic administration.^[^ ^80^, ^81^ ^]^ Additionally, DFO's efficacy can be enhanced via alternative delivery strategies. For instance, it can be administered through targeted delivery methods or intranasal routes to bypass BBB limitations.^[^ ^82^ ^]^ Moreover, DFO's ability to reduce the systemic iron available for transport across the BBB presents a therapeutic strategy as an iron deficiency inducer for GBM treatment.^[^ ^83^ ^]^ These considerations highlight the importance of delivery mechanisms in the translational application of iron chelators for GBM therapy.

Further research is needed to elucidate the underlying mechanisms responsible for these observations and to identify novel therapeutic modalities for GBM treatment. Additionally, further investigation on understanding adaptive mechanisms in chemo‐resistant cells due to the complex interplay between different signaling pathways in response to drugs is required. We should note that a more sophisticated 3D model of GBM microenvironment, including other cells such as tumor‐associated macrophages (TAMs), can further elucidate the role of inter‐ and intra‐tumoral interactions on GBM response to iron‐chelators.^[^ ^84^, ^85^ ^]^

In summary, iron and oxygen are important cellular resources for metabolism, growth, and survival of cancer cells. Their roles in cancer therapeutics are complex and multifaceted. TMZ‐resistant cells have higher iron levels and enhanced RRM2, making them vulnerable to iron chelation and offers a potential approach in cancer therapy. On the other hand, hypoxia presents challenges in cancer treatment. Cancer cells in hypoxic regions are more resistant with altered drug metabolism. Here, we studied the antitumor effects of chelator induced iron deficiency, on non‐resistant versus TMZ‐resistant GBM in 2D and 3D models with the aim of breaking TMZ resistance in these cells. To better understand the mechanisms that lead to iron chelation‐induced cell death, we monitored several downstream outcomes. We observed iron chelators reduced viability and proliferation, up‐regulated HIF1‐α expression, increased ROS generation, and induced autophagy. The results did not confirm predominant mitochondrial apoptosis. The CI index values obtained for the co‐treatment of non‐resistant and TMZ‐resistant tumoroids, generated from cell lines and patient‐derived cells, with ranges of TMZ + DFO or DFP showed potential synergistic effects. The combined therapy also induced a significant reduction in the size and the relative invasion length of the tumoroids. Results of this study suggest that iron chelators are promising drug candidates to improve therapeutic treatments of chemo‐resistant cells. Due to the chemoresistance of hypoxic and stem‐like cells, the manipulation of iron in these cells offers potential avenues to improve the efficacy of cancer treatments. However, cancer is a heterogeneous disease, and therapies targeting iron and oxygen pathways may be more effective in specific cancer types or stages. Therefore, the intricacies of these processes necessitate more research on their potential for novel cancer treatments. We should note that, although GBM cells exhibit elevated iron levels which reduces the efficiency of TMZ, excessive iron depletion may disrupt essential functions and lead to systemic side effects. However, many years of clinical iron chelator therapy with DFO has created a very well‐known window of therapeutic efficacy for DFO and DFP.^[^ ^86^, ^87^ ^]^ Additionally, a balanced therapeutic strategy, such as combining iron chelators with chemotherapy, can achieve controlled iron modulation, optimizing the therapeutic window for effective GBM treatment. Iron depletion therapy must be carefully managed to avoid inducing hypoxia and subsequent upregulation of hypoxia‐inducible factor 1‐alpha (HIF‐1α), which can promote chemoresistance and tumor recurrence in glioblastoma (GBM). While DFO and DFP are FDA‐approved for treating iron overload conditions, their use in cancer therapy requires close monitoring to balance iron levels and minimize hypoxia‐related complications.^[^ ^82^ ^]^ Although studies indicated that iron chelation disrupts metabolic pathways essential for tumor survival, excessive iron depletion may exacerbate hypoxic stress,^[^ ^88^ ^]^ targeting iron metabolism in combination with therapies addressing the hypoxic microenvironment, such as hypoxia‐activated prodrugs or HIF‐1α inhibitors, could mitigate these effects.^[^ ^89^ ^]^ Additionally, hypoxia can present new opportunities to identify synthetic lethal phenotypes. For instance, DFO may sensitize cells to therapies that target hypoxia‐related vulnerabilities, such as anti‐angiogenics like Bevacizumab, CA‐IX inhibitors, or glutaminase inhibitors. Therefore, the variability in tumor biology and metabolic states underscores the importance of personalized treatment regimens to optimize therapeutic outcomes while minimizing adverse effects.^[^ ^90^ ^]^

Pharmacokinetic compatibility is another critical consideration for optimizing the therapeutic efficacy of the combination therapy. TMZ, an oral alkylating agent, achieves peak plasma concentrations within 1–2 h and has a half‐life of ≈1.5 h, with minimal interactions involving cytochrome P450 pathways.^[^ ^91^, ^92^ ^]^ In contrast, DFO and DFP, administered intravenously and orally, respectively, exhibit distinct pharmacokinetics, with DFO having a shorter half‐life (2–4 h) and DFP providing more sustained iron chelation.^[^ ^93^, ^94^ ^]^ The combined use of these drugs necessitates careful timing and dosing to avoid potential competition for absorption or changes in drug transport mechanisms caused by systemic iron modulation. Iron chelation may influence TMZ distribution and elimination, while increased oxidative stress due to ROS generation by iron chelators could further affect TMZ's pharmacodynamics.^[^ ^95^ ^]^ To address these challenges, precise dosing regimens and pharmacokinetic modeling are essential. Humanized models, such as multi‐organ‐on‐chip systems, followed by animal studies, could provide valuable insights into optimizing combination therapy for maximum efficacy and minimal toxicity.

It is important to consider that, in animal models, the tumor microenvironment's hypoxic and nutrient‐variable conditions amplify differences in iron metabolism between TMZ‐resistant and TMZ‐sensitive cells.^[^ ^96^ ^]^ Tumor‐associated macrophages enhance iron uptake by GBM cells, while systemic iron regulation introduces dynamic iron availability, allowing resistant cells to adapt their uptake and export mechanisms for survival.^[^ ^97^, ^98^ ^]^ Additionally, resistant cells exhibit stronger antioxidant defenses and iron‐export mechanisms, enabling them to manage iron‐induced ROS more effectively than sensitive cells in vivo.^[^ ^99^ ^]^ These adaptations enhance their survival and invasiveness in the complex in vivo environment, highlighting the need for therapies that specifically target these adaptive mechanisms. Additionally, iron chelators such as deferoxamine (DFO) and deferiprone (DFP) have shown anticancer potential in animal models across various cancers.^[^ ^31^, ^100^ ^]^ For instance, DFO demonstrated dose‐dependent antiproliferative effects in leukemia and induced apoptosis in mammary adenocarcinoma models.^[^ ^101^ ^]^ Iron chelators like VLX600 have also been shown to inhibit mitochondrial energy production, targeting both proliferative and quiescent tumor cell populations.^[^ ^102^ ^]^ In the context of GBM, suppression of the homeostatic iron regulator (HFE) gene reduced tumor cell growth, underscoring the potential of iron‐targeted approaches.^[^ ^103^ ^]^ Although in vivo studies specifically exploring the combination of iron chelators with TMZ in TMZ‐resistant GBM are limited, the data suggests that iron chelators may enhance therapeutic effects by disrupting iron‐dependent pathways critical for tumor survival and repair mechanisms.

There are currently ongoing in vivo investigations on enhancing the therapeutic effects of iron chelators in GBM and leptomeningeal metastases (LM). For instance, in LM mouse models, DFO reduced cerebrospinal fluid (CSF) iron, diminished LM growth, and prolonged survival.^[^ ^104^ ^]^ A clinical trial (NCT05184816) is investigating DFO's efficacy in LM, with promising preliminary results. Other trials include those targeting triple‐negative breast cancer (NCT05300958) and hepatocellular carcinoma (NCT03652467). Iron chelators with radiation shows promise in animals with GBM.^[^ ^105^ ^]^ There are emerging strategies in targeting iron uptake and depletion. For instance, transferrin receptor 1 (TfR1) is a promising therapeutic target due to its elevated expression in GBM.^[^ ^106^ ^]^ Novel agents like Gallium nitrate and newer iron chelators, including VLX600 and Dp44mT, have shown efficacy. In a GBM xenograft model, Dp44mT was found to have high efficacy.^[^ ^107^ ^]^ Additionally, iron chelation in a mouse intracranial hemorrhage model shifted microglial polarization toward an anti‐inflammatory state.^[^ ^108^ ^]^ Further research is needed to improve the precision, selectivity, and effectiveness of iron chelators for brain tumors while addressing limitations such as resistance mechanisms and metabolic challenges. These advancements hold promise for personalized medicine approaches to GBM treatment.

## 4 Experimental Section

### Drugs, Reagents, and Equipment

A hypoxic incubator chamber (Cat# 27 310) and a single flow meter (Cat# 27 311) were purchased from Stemcell Technologies. Pre‐mixed gas of 2% O_2_, 5% CO_2_ and 93% N_2_ (part # NI CD5O2C‐Q) was provided by Linde Canada, Inc. Glucose oxidase (GOD, CAS# 9001‐37‐0), peroxidase (POD, CAS# 9003‐99‐0), dextrose (CAS# 50‐99‐7), temozolomide (TMZ, CAS# 85622‐93‐1), deferoxamine (DFP, CAS# 30652‐11‐0), deferiprone (DFO, CAS# 138‐14‐7), 2′, 7′‐dichlorodihydrofluorescein diacetate (DCFH‐DA, CAS# 4091‐99‐0), trypan blue (CAS# 72‐57‐1), presto blue (CAS# A13262) and trypsin‐EDTA solution (P# T4049) were purchased from Sigma Aldrich.

Potassium iodide (CAS# 7681‐11‐0) from Caledon laboratory, Citric acid monohydrate (CAS# 5949‐29‐1) and sodium citrate (CAS# 6132‐04‐3) from Bio basic Canada Inc. RFP‐GFP tagged adenovirus was donated by Ghavami's lab.

Human glioblastoma cell line (h U251) was purchased from ATTCC (Masassas, VA, USA). Dulbecco's Modification of Eagle's Medium, high glucose, L‐glutamine (DMEM, Cat# 11 965 092), DMEM, no glucose, no glutamine, no phenol red (Cat# A1443001), Fetal Bovine Serum (FBS, Cat# 10 437 036), penicillin‐streptomycin (P/S Cat# 15 140 122), HIF1‐α Monoclonal antibody (Mgc3) PE‐conjugated (Cat# 12‐7528‐82), mouse IgG1 kappa isotype control PE (Cat# 12‐4714‐41), protease inhibitor cocktail (Cat# 87 785), pierce BCA protein assay kit (Cat# 23 227) and page ruler protein ladder (Cat# 26 620) were purchased from Thermofisher.

NuPage 4–12% Bis‐Tris gels, 1.0 mm, 10‐well (Cat# NP0321PK2), NuPage 10× Sample Reducing Agent (Cat# NP0009) and NuPage 4× LDS Buffer (Cat# NP0008) were purchased from Invitrogen. Intercept (PBS) blocking buffer (Cat# 927–70001) was purchased from Licor. Rabbit anti LC3B (Cat# 100–2220) was bought from Novus. Rabbit anti BAX (Cat# 92 772), rabbit anti caspase3 (9662), mouse anti HIF1‐α (Cat# 79 233), rabbit anti β‐actin (Cat# 4967), anti‐rabbit IgG (H+L)‐DyLight 800 (Cat# 5151) and anti‐mouse IgG (H+L)‐DyLight 800 (Cat# 5257) were purchased from CellSignaling. Nitrocellulose membrane was purchased from Pall (product ID# 66 485).

Semi‐dry Transfer Buffer was made in house with mixing 11.64 g of 48 mM Tris (Cat# BDH4500, VWR), 5.86 g of 39 mM glycine (Cat# BP38150, ICN), 7.5 mL of 0.037% SDS (Cat# L5750, Sigma) and 20% methanol (Cat# MX0485‐3, EMD) with 2L H2O.

### Glucose Assay

2 × 104 cells were cultured in 24 well plates with DMEM supplemented with 10% (v/v) Fetal Bovine Serum (FBS) and 1% (v/v) Penicillin/Streptomycin, incubated at 37 °C in 5% CO_2_ and 95% air. When cells were 50–60% confluent, the media of wells were removed and media with desired concentrations of glucose, 0.025 to 1 mg mL^−1^, were added to each well. The rate of change in the concentration of glucose in each well were monitored over time (10 h) using glucose oxidase assay with colorimetric method, following the manufacturer's protocol. In this method, the glucose oxidase enzyme (GOD) catalyzes the oxidation of glucose to hydrogen peroxide (H_2_O_2_) and peroxidase (POD) reaction was then used to calorimetrically visualizing the formed H_2_O_2_.^[^ ^30^ ^]^

Additionally, the cell count in each well was used to normalize the uptake rate. It was observed that the uptake rate remains constant above a certain threshold, but below this threshold, the rate becomes dependent on the glucose concentration. To measure the proliferation rate, the number of cells before adding desired concentrations and after 24 and 48 h of that were counted for each concentration using Trypan blue assay.

To measure the rates of glucose uptake and proliferation of hypoxic cells, cultured cells were transformed into a hypoxic chamber (Stemcell Technologies; Cat# 27 310) filled with a pre‐mixed gas of 2% O_2_, 5% CO_2_ and 93% N_2_. After 24 h the same glucose measurement and Trypan blue assays were used to measure the rates of glucose uptake and proliferation.

### Rate of Proliferation

After the indicated treatment times, cells were trypsinized, dissociated and centrifuged in 300 g for 5min. The pallet was resuspended, and 0.4% trypan blue solution was mixed in 9:1. 10 µL of the solution was added to the hemocytometer, and number of live/dead cells was counted using immunofluorescence (IF) microscope.

### Immunofluorescence Imaging

Accumulation of HIF1‐α was imaged using HIF1‐α alpha antibody. U251 non‐resistant and TMZ‐resistant cells were grown on coverslips (5 × 10^4^ cells per 35 mm) and treated with DFO and/or placed in hypoxic chamber. After 24 h, cells were fixed using 90% methanol for 15 min in −20 °C and stained with HIF1‐α antibody according to the company's protocol. After 2 h of incubation at room temperature, cells were imaged using IF microscope.

Flow cytometry was used for semi‐quantification analysis. To prepare the cells for flow cytometry, cells were washed in PBS, trypsinized with trypsin‐EDTA, resuspended in culture media, centrifuged at 400 g for 3 min, the pallet was resuspended in 90% methanol and incubated in −20 °C for 15 min. All the washing and dissociation steps were done on ice in presence of 50 µM DFO to inhibit HIF1‐α degradation. Methanol was then neutralized with excess amount of PBS and cells were washed and centrifuged. Cell pallet was resuspended in PBS and a HIF1‐α antibody was added according to the company's protocol. To remove non‐specific interactions, isotype antibody was used as negative control of the experiment.

### Cell Lines and Culture

Human glioblastoma multiform cells (U251) were cultured in DMEM with 10% (v/v) FBS and 1% (v/v) PS and maintained in 5% CO_2_ and 95% air at 37 °C in humidified standard cell culture incubator. Culture medium was replaced every other day. Monolayer cells were trypsinized, centrifuged at 300 g for 5 min, supernatant was removed, and the pallet was resuspended in fresh media. To count the cells, 0.4% trypan blue solution were mixed in 9:1 and 10 µL of the solution was added to the hemocytometer. Number of live/dead cells was counted using microscope. The IC50 of cells was measured (111 µM) and verified.^[^ ^109^ ^]^

### 2D Culture

In all experiments, non‐resistant and TMZ‐resistant U251 cells were seeded at a concentration of 5 × 10^5^ cells mL^−1^ in 10 mL volumes into 100 mm Petri dishes. These cells were maintained in complete high glucose DMEM media, with the non‐resistant cells in TMZ‐free media and the resistant cells in media containing 250 µM TMZ. The cells were incubated at 37 °C and 7.5% CO_2_ in a humidified incubator for 4 days. After incubation, cells were harvested by scraping and washed with PBS. The resulting cell pellets were stored in a PBS buffer containing a 1:75 ratio of phosphatase inhibitor cocktail (Sigma, Cat#P5726) and protease inhibitor cocktail (Sigma, Cat#P8340) at −80 °C until further analysis.

### Proteomics Data Analysis

The mass spectrometry proteomics data have been submitted to the ProteomeXchange Consortium via the PRIDE^[^ ^110^ ^]^ partner repository with the identifier PXD037753. Raw files were created using XCalibur 4.2.28.14 (Thermo Scientific). These Thermo raw files were processed with MaxQuant (V.1.6.2.10), and MS/MS spectra were examined against the Uniprot (Swiss‐Prot) protein sequence database (Homo Sapiens, release May 2021) using Andromeda, setting an FDR for identification at 0.1. The MaxLFQ algorithm was employed to quantify proteins. Database search parameters included protein N‐terminal acetylation and deamidation, methionine oxidation as variable modifications, and cysteine carbamidomethylation as a fixed modification. This resulted in the identification of 4122 proteins, which were then analyzed using Perseus (V.1.6.2.2). Data filtering removed reverse sequence proteins and common lab contaminants, resulting in 3879 proteins. For Principal Component Analysis (PCA) and Hierarchical Clustering Analysis (HCA), proteins not associated with TMZ resistance were excluded following ANOVA analysis, retaining 1100 proteins with Z‐score normalized expression values. In PCA, normalized values were plotted using a Benjamini‐Hochberg threshold of 0.05, utilizing Perseus's built‐in tool. HCA, based on Euclidean distance, assessed group similarities.

Data filtering ensured at least 70% valid values in each group. The non‐normalized values of 2024 identified proteins were log_2_ transformed, with missing values replaced by −1. Differentially expressed proteins were identified using a two‐tailed t‐test with 250 randomizations, setting FDR at 0.05 and S0 at 0.1. Results were visualized in volcano plots, and data were exported to JMP 16.0.0 for further analysis. Upregulated proteins were marked in red, and downregulated ones in blue.

Proteomics data were retrieved from the ProteomeXchange Consortium/PRIDE repository (PXD017952) for comparative analysis with primary and recurrent human tumors, analyzed in MaxQuant (V.1.6.8.0) with an FDR of 1% at the PSM, peptide, and protein levels. Spectra were matched to human protein sequences from Uniprot (Homo Sapiens, release May 2021) and quantified using the MaxLFQ algorithm with at least two unique or razor peptides. The database search parameters were as previously described. For MaxQuant, precursor mass tolerance was 20 ppm, and fragment ion mass tolerance was 0.15 Da, with a minimum peptide length of 7 amino acids and up to two missed cleavages allowed. A combined matrix of common protein expressions in resistant and non‐resistant cells/spheroids and primary and recurrent tumors (1191 proteins) was created and analyzed in Perseus (V.1.6.2.2). Contaminants, reverse identifications, and site‐only proteins were excluded.

Samples were grouped and filtered for a minimum of 70% valid values per group (1007 proteins). Non‐normalized expression values were log2 transformed and missing values imputed based on a normal distribution (width 0.3, downshift 1.8). Log2 values were Z‐scored and hierarchically clustered. Normalized data were exported to MetaboAnalyst 5.0 for visualization. Heatmaps were manually curated to exclude clusters not involved in TMZ resistance.

Volcano plot analysis compared TMZ‐resistant and non‐resistant cells in 2D and 3D cultures to identify protein‐level differences, indicating significantly (q < 0.05) downregulated (log_2_ fold change ≤ −0.5) or upregulated (log_2_ fold change ≥ 0.5) proteins. Proteins with over twofold regulation were annotated.

### Establishment of TMZ‐Resistant Cells

U251‐mKate cells, developed by Dr. Marcel Bally from the British Columbia Cancer Agency in Vancouver, BC, Canada, were cultured in T75 tissue culture flasks with high glucose DMEM (Gibco; Cat #: 11 965 092), supplemented with 10% FBS (Gibco; Cat #: 10437‐036) and 1% Penicillin‐streptomycin (Gibco, Cat#:15 140 122). The cells were incubated at 37 °C in a humidified environment with 7.5% CO_2_ (Thermo Fischer Scientific). To create a chemo‐resistant model, we employed a pulsed‐selection approach.^[^ ^111^ ^]^ Upon reaching 70–80% confluence, the cells were treated with 100 µM TMZ (Sigma‐Aldrich, CAS #:85622‐93‐1) for 3 weeks, followed by a 4‐week period in TMZ‐free media for recovery. This was followed by culturing the cells in complete media containing 250 µM TMZ for 3 weeks, then allowing another 4 weeks of recovery in TMZ‐free media. The final population of U251‐mKate cells that developed resistance to 250 µM TMZ was subsequently maintained in complete media without TMZ for an additional 4 weeks.

### Cell Viability

1 × 10^4^ non‐resistant and TMZ‐resistant cells per well were seeded in 96 well plate. After 48 h, three concentrations (10, 50 and 100 µM) of DFO and DFP were added to each well. Cells were incubated either in culture incubator (normoxia) or hypoxic chamber. After 24, 48, and 72 h, cell media was removed from each well and 10% presto blue solution was added. Cells were incubated for 20 min; the supernatant was then collected, and fluorescent intensity was measured at 560/590 nm ex/em.

### Autophagy Flux Assay

To monitor autophagosomes, autophagolysosomes, and autophagy flux we monitored the localization of LC3 using GFP‐RFP‐LC3 adenovirus. In brief, U251 non‐resistant and TMZ‐resistant cells were grown on coverslips (50 000 cells per 35 mm) and treated with three concentrations (10, 50, and 100 µM) of DFO and DFP. After 24 h, cells were transfected (50 MOI) with GFP‐RFP‐LC3 adenovirus. 24 h after transfection, cell medium was removed, and coverslips were rinsed in PBS. Also, cells were post‐treated with 750 nM Rap and 750 nM Rap + 30 µM CQ as the positive controls for the experiment. Autophagic flux was visualized by imaging autolysosomes (red puncta) in cells treated with 750 nM Rapamycin (Rap), while inhibition of autophagic flux was monitored by co‐localization of mRFP/GFP (yellow puncta) in 750 nM Rap + 30 µM CQ‐treated cells.

### Immunoblotting

U251 non‐resistant and TMZ‐resistant cells were seeded on a 3 cm Petri‐dishes. After 48 h, they were split into two groups; cells in the first group were kept in cell culture incubator and the other group was incubated in hypoxia. After 24 h, three concentrations (10, 50, and 100 µM) of DFO and DFP were added to each Petri dish. To extract protein from the cells, after 24 h and 72 h of treatments, cell media were removed, cells were washed twice with cold PBS and 400 µL of cold RIPA lysis buffer (with protease inhibitor) was added to each Petri dish (all steps were performed on ice). Cells were then gently detached from the dish using a cell scraper. The resulting cell lysates were transferred to 1.5 mL tubes and repeatedly pipetted 20 times to homogenize and facilitate protein extraction. The lysates were then allowed to incubate for 15 min at 4 °C and centrifuged at 14 000 g for 10 min at 4 °C to remove cell debris. Protein concentration of each lysate was determined with a Pierce BCA protein assay kit (Thermo Fisher). After that, each lysate was mixed with Sample Reducing Agent (Thermo Fisher) and LDS Sample Buffer (Thermo Fisher), and double‐distilled water (ddH_2_0) and adjusted to a final concentration of 2.5 mg mL^−1^. The resulting lysate master mixes were heated at 95 °C for 10 min and stored at −20 °C until performing western blot. Four independent gel electrophoresis were performed in MES buffer (Thermo Fisher) using precast 15 mm 4–12% bis‐tris gel (Thermo Fisher). Specifically, gel 1 and 3 were used to assess Bax and HIF‐1α and gel 2 and 4 were used to assess Caspase 3 and LC3b. For all four gels, we collected lysates from the same batch of treated cells and prepared a single lysate master mix. Based on the BCA quantification, a total of 40 µg of protein (in 16 µL) for each sample was loaded in each lane and for each gel. After gel electrophoresis, proteins in each gel were semi‐dry transferred onto a nitrocellulose membrane (22 V for 45 min). The membranes were then blocked for 60 min in Intercept (PBS) Blocking Buffer (LI‐COR Biotech), cut and blotted using indicated primary antibodies overnight at 4 °C. Due to the proximity of the molecular weight for β‐actin (45 kDa) and Caspase 3 (35 kDa), we elected not to cut or probe for β‐actin in gel 2 and 4 given that the exact same volume and amount of protein was loaded into gel 2 using the same master mix in gel 1 and that the exact same volume and amount of protein was loaded into gel 3 using the same master mix in gel 4. After primary antibody incubation, the membranes were washed three times in PBST (PBS containing 0.1% Tween 20) and incubated in blocking buffer containing secondary antibodies (anti‐rabbit IgG DyLight 800 4× PEG conjugate, Cell Signaling, 1:30 000) for 60 min at room temperature. Finally, the membranes were washed three times in PBST and rinsed three time in PBS before being scanned using an Odyssey Infared Imager (LI‐COR Biotech). The primary antibodies and their dilutions are as follows: Rabbit anti Bax (Cell Signalling 1:1000); Rabbit anti HIF‐1α (Cell Signalling 1:1000); Rabbit anti Caspase 3 (Cell Signalling 1:1000); LC3b (Novus Biologicals 1:1000).

### Ferrozine Assay

TMZ‐resistant and non‐resistant U251 cells were seeded on 6 or 12 well plates and cultured until confluent. Cells were then treated with media or one of three doses of deferiprone or deferoxamine (10, 50, or 100 µM) for 24 h. After treatment, the cells were rinsed several times with PBS and stored on the plate at −20 °C until analyzed.

To determine intracellular iron, a ferrozine‐based assay was utilized as per,^[^ ^35^, ^36^ ^]^ and Reimer, et al., with some modifications. Briefly, the cell samples were lysed directly in well plates with a sufficient volume of Cell‐lytic M lysis buffer (containing 1% protease inhibitor cocktail III and 1 mM PMSF) to cover cells. Plates were placed on an Infors HT Ecotron orbital shaker (Bottmingen, Switzerland) for 30 min (100 RPM, 25 °C), then transferred to microcentrifuge tubes. A small aliquot of 10 µL was taken for protein analysis. The remaining volume of lysate was dried at 95 °C in a covered VWR dry block heater (Dublin, Ireland). Cell lysates were rehydrated with 75 µL of ddH2O and 75 µL 40 mM HCl. To this solution, 37.5 µL of 1.4 M HCl and 4.5% w/v KMnO_4_ was added sequentially, then lysates were digested at 60 °C in a VWR dry block heater. Samples were cooled, then 22.5 µL of iron detecting reagent containing 6.5 mM neocuproine, 6.8 mM ferrozine, 2.5 mM ammonium acetate and 1.0 mM ascorbic acid was added. Samples were incubated at 28 °C for 30 min in the dark, then centrifuged at 16 000 G for 5 min to remove any remaining cell debris. The supernatant was plated into a 96‐well plate and read at 550 nm on a multilabel plate reader. To prepare a 10‐point standard curve, a range of iron standards from 0.6 to 150 µM were prepared using serial dilution from 300 µM FeCl_3_ in 10 mM HCl. A 75 µL volume of ddH_2_O was added to 75 µL of iron standard, then these samples were treated identically to cell lysates.

A protein assay was performed on 10 µL of cell lysate using Pierce BCA Protein Assay Kit (23 225, Thermofisher) and following manufacturer's instructions. Intracellular iron content was normalized to protein content.

### ROS Assay

TMZ‐resistant and non‐resistant U251 cells were seed into 96‐well plates. After 48 h, cells were categorized into two groups, hypoxic and normoxic cells. The former was kept in hypoxic chamber and the latter was maintained in cell culture incubator. After 24 h, both groups were treated with three concentrations (10, 50, and 100 µM) of DFO and DFP. After 24 h and 72 h of treatment, cells were rinsed twice with PBS to remove any traces of media. 100 µL of 10 µM DCFH‐DA were added to each well. The plate was protected from light and incubated on a plate shaker with gentle agitation for 30 min at 37 °C. Fluorescent intensity was measured at 488/535 nm ex/em.

### Real‐Time PCR

β‐subunit (R2) is a homodimer encoded by the RRM2 gene and combines with RRM1 to allow for assembly of the heterodimeric tetramer, ribonucleotide reductase (RNR), which produces dNTPs for nuclear DNA replication and repair. RNR consists of different subunits including RRM2, which is a usual subunit measured for the expression of RNR. TMZ‐resistant and non‐resistant U251 cells were seeded into 6‐well plates and were kept in normoxia and hypoxia. After 24 h of treatment with DFO and DFP, they were wash with ice‐cold PBS and dry pellet stored at −20 °C. mRNA extraction and DNase digestion were carried out following the protocols provided with Qiagen's RNeasy Mini Kit and RNase‐Free DNase kit. The purity of the extracted RNA was evaluated using a Nanodrop spectrophotometer. First‐strand cDNA synthesis was performed using the SuperScript III First‐Strand Synthesis System from Invitrogen, with cDNA purity also checked via Nanodrop. The QuantiNov SYBR Green PCR Kit was then utilized to set up reactions for the quantitative analysis of RRM2. The following primers were purchased from IDT;

RRM2: 5′‐GCGATTTAGCCAAGAAGTTCAGAT‐3′ (forward)
and 5′‐CCCAGTCTGCCTTCTTCTTGA‐3′ (reverse),
β‐actin: 5′‐ATCTGGCACCACACCTTCTA‐CAA‐3′ (forward)
and 5′‐GTACATGGCTGGGGTGTTGAAG‐3′ (reverse).

The fold change of mRNA expression was calculated based on the relative gene‐expression quantification method according to the comparative C_t_ method where β‐actin was used as an endogenous control; 2^−(ΔCt sample‐ΔCt control)^.^[^ ^112^ ^]^ ΔC_t_ is the difference between the Ct values of the target gene and the β‐actin.

### Tumoroid Generation on‐a‐Plate

The formation of U251 multicellular tumoroids was achieved using Apricell Biotechnology's hydrogel‐based microfluidic‐integrated culture plate (3‐in‐1 plate) with U‐shaped microwells. Hydrogel inserts were created by replica molding the 3D‐printed microfeatures. The inserts were placed in 6‐well plates and gently seeded with 100 µL of culture media containing 2 × 10^5^ cells in the seeding zone. The wells were then incubated at 37 °C for 10 min, allowing the cells to migrate into the microwells through the microchannels. After incubation, 300 µL of fresh culture media was carefully added to the media reservoir to cover the cells in the microwells. The growth of the U251 tumoroids was monitored daily over a period of 3 days.

### ECM Embedding and Combinatorial Drug Testing

To resemble the tumor ECM conditions of glioblastoma, bovine fibrillar collagen at a concentration of 4 mg mL^−1^ was employed as the primary component of the extracellular matrix (ECM). To prepare the hydrogel solution, the pH and ionic concentrations were adjusted by adding 10× PBS and 0.5 N NaOH to the collagen stock solution in a 1:1:8 ratio. Next, an appropriate volume of culture media was immediately mixed with the collagen solution to achieve the desired concentration. The final collagen mixture was gently introduced into the ECM loading zone of the culture plate insert and incubated at 37 °C for 45 min to form a gel.

Various concentrations of DFP and DFO (ranging from 10 to 100 µM) were administered in a single treatment setting to investigate the impact of iron‐chelating drugs on the metabolic activity and cytotoxicity of brain tumoroids. 4‐day‐old tumoroids were treated with these drugs by first aspirating the primary culture medium from the media reservoir, then adding 300 µL of fresh media containing the drugs. For combination drug treatment, different concentrations of TMZ (50, 100, 250, and 500 µM) were added following the iron‐chelating drug treatment on the tumoroids.

### Drug Toxicity and Live/Dead Staining

To determine the viability of tumoroids, a Live/Dead assay was utilized, employing 1 µM calcein AM and 4 µM ethidium homodimer‐1 (Life Technologies kit) for 30 min at 37 °C. The entire ECM‐embedded tumoroid model was stained and imaged directly within the culture plate insert, avoiding the need to extract the tumoroids before staining. The influence of drug treatments, both individually and in combination, was evaluated through fluorescent imaging and quantification of the cells that invaded the ECM.

To assess the cytotoxic effects of various drug concentrations and exposure durations on the tumoroid cells, the Presto Blue assay was applied. This assay gauges the metabolic activity of viable cells through fluorescence, with excitation and emission wavelengths set at 560 nm and 590 nm, respectively. A solution containing 10% Presto Blue reagent, relative to the total volume of the culture medium, was directly added to the media reservoir of the insert, ensuring it covered the tumoroids during a 3‐h incubation. The average fluorescence intensity of the tumoroids was then measured and corrected by subtracting the intensity values from blank, cell‐free microwells.

### Synergy Analysis

The viability data were entered into CompuSyn software to analyze the combinatory impact of cotreating with TMZ and either DFO or DFP. This analysis was performed using the combination index (CI) method developed by Chou and Talalay.^[^ ^113^ ^]^ The results were interpreted as follows: a CI index below 1 indicated a highly synergistic effect, a CI index between 1 and 2 signified a non‐synergistic or additive effect, and a CI index above 2 suggested an inhibitory effect.

### Statistical Analysis

All data are presented as means ± SD. Statistical significance of differences was determined using GRAPHPAD PRISM 5.0 software (Graphpad, San Diego, CA, USA) through one‐way or two‐way ANOVA, followed by Bonferroni's post hoc test. A P‐value of less than 0.05 was considered statistically significant. A two‐tailed P‐value of less than 0.05 was deemed to indicate a statistically significant correlation. Unless stated otherwise, experiments were conducted independently three times.

## Conflict of Interest

Authors declare that they have no competing interests.

## Author Contributions

M.A., M.A., and P.W. developed the concept and the methodology. M.A., M.A., P.W., and J.L performed analysis and interpretation of data. M.A., A.S. and S.S., S.L., T.Z. and M.S. performed the experiments. M.A. performed the biological data analysis. M.A. and P.W. prepared the original draft. All authors contributed to reviewing and editing the manuscript.

## Supporting information



Supporting Information

## Data Availability

The data that support the findings of this study are available from the corresponding author upon reasonable request.
