# Correction to “Tumoroid Model Reveals Synergistic Impairment of Metabolism by Iron Chelators and Temozolomide in Chemo‐Resistant Patient‐derived Glioblastoma Cells”

**DOI:** 10.1002/advs.202519458

**Published:** 2025-11-19

Amereh M, Seyfoori A, Shojaei S, Lane S, Zhao T, Shokrollahi Barough M, Lum JJ, Walter PB, Akbari M.

Adv Sci (Weinh) 2025 May;12(20):2412505.

https://doi.org/10.1002/advs.202412505


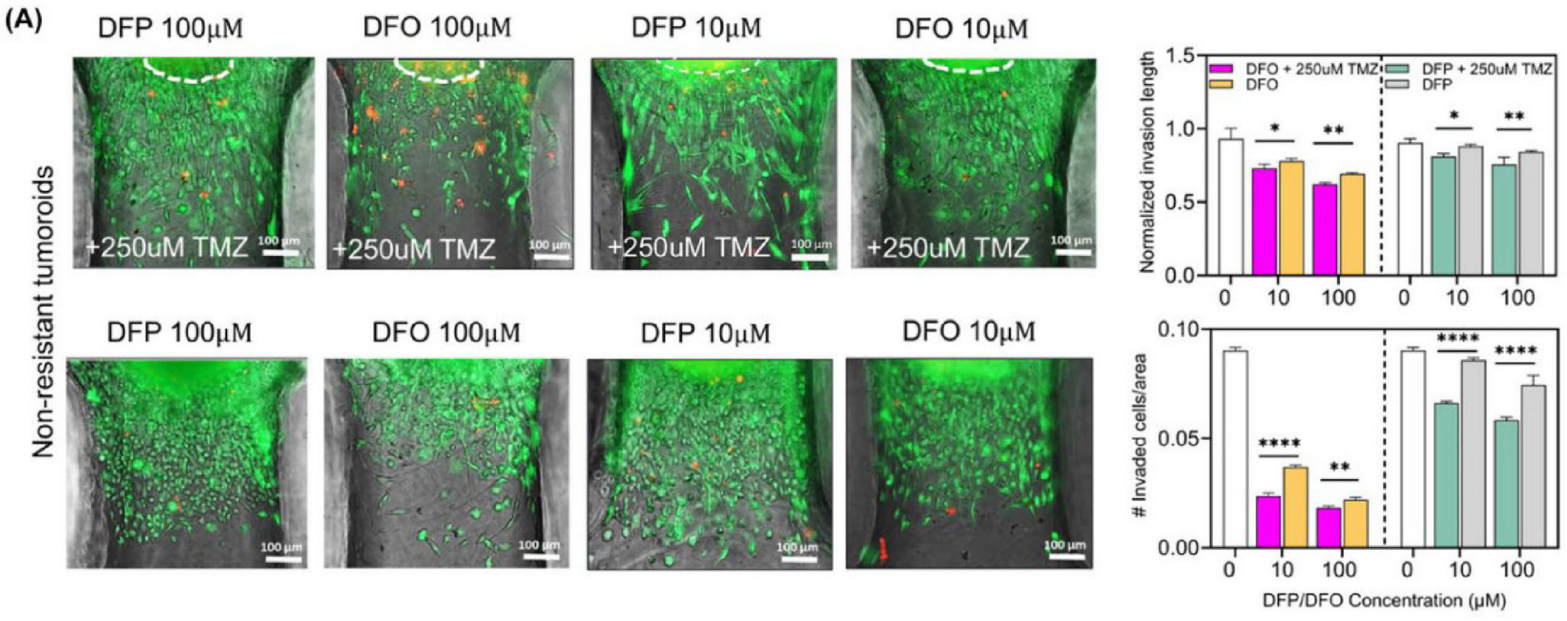


Figure 3A. (second row, DFO10µM condition) above is incorrect. This error occurred during the final stages of preparation of the manuscript, and despite our efforts to identify it, we unfortunately missed it. We would like to clarify that the images are representatives from different treatment groups and the misplacement of the figure does not affect the study's conclusions in any way. We kindly ask for Figure 3A to be corrected as follows:

Corrected Figure 3A:


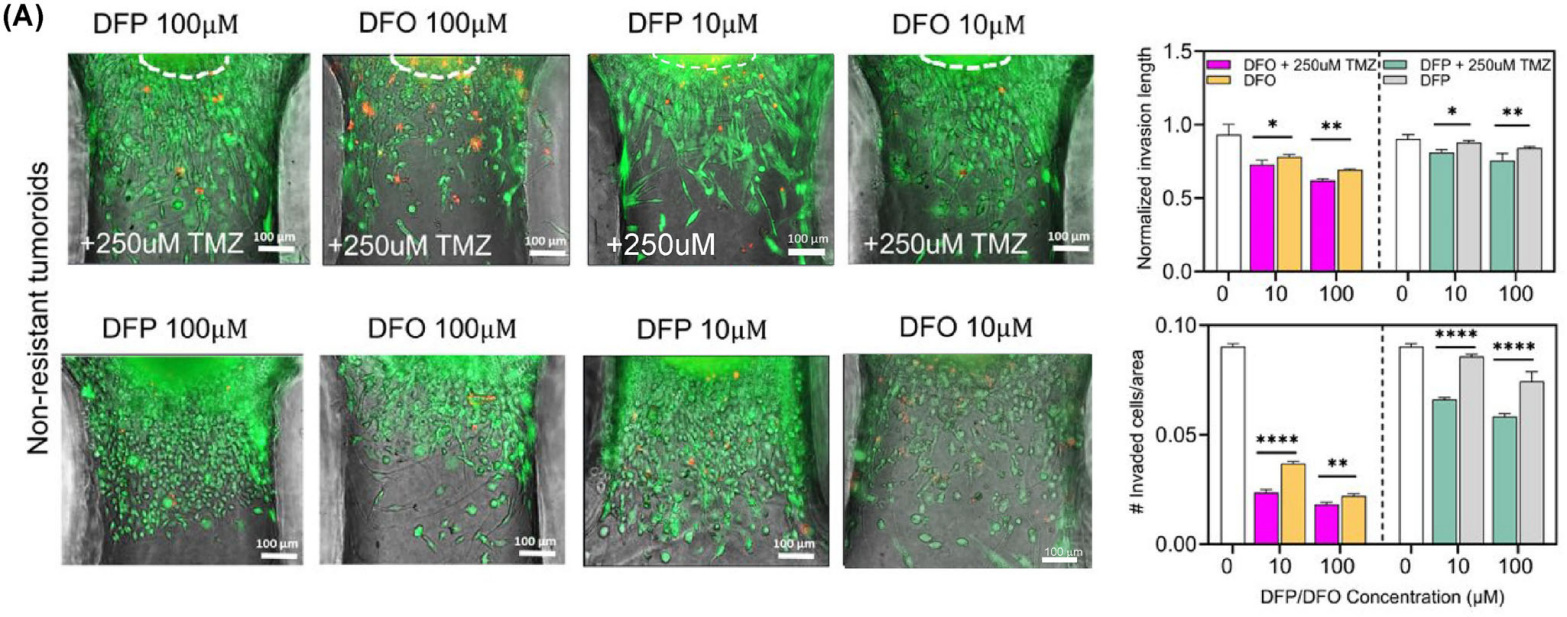


We sincerely apologize for these errors.

