# Supplementary material for: Tumoroid Model Reveals Synergistic Impairment of Metabolism by Iron Chelators and Temozolomide in Chemo‐Resistant Patient‐derived Glioblastoma Cells
Source: Adv Sci (Weinh). 2025 Apr 26;12(20):2412505. doi: 10.1002/advs.202412505 (PMC12120723; doi:10.1002/advs.202412505)
Supplement: Supplementary file 1 — Supporting Information [file ADVS-12-2412505-s001.docx]

**Supplementary Materials**

Characterization of the molecular signature of TMZ-resistant vs. non-resistant GBM in 2D and 3D cultures (Fig. S1) using proteomics and secretome analyses.

**Figure S1. Characterization of human glioblastoma (hGBM), TMZ-resistant vs. non-resistant using proteomics and secretome analysis.** A schematic of a brain tumor and surrounding tissue. (A) Formation of non-resistant and TMZ-resistant tumoroids using the EZ-Seed culture platform. (B) Tumoroids resided within a hydrogel matrix mimicking the brain tumor niche in tumor microenvironment (TME). (C) Workflow of the study. Steps on recapitulating resistant human glioblastoma and characterization of resistant tumoroids using proteomics, and (D) secretome analyses.


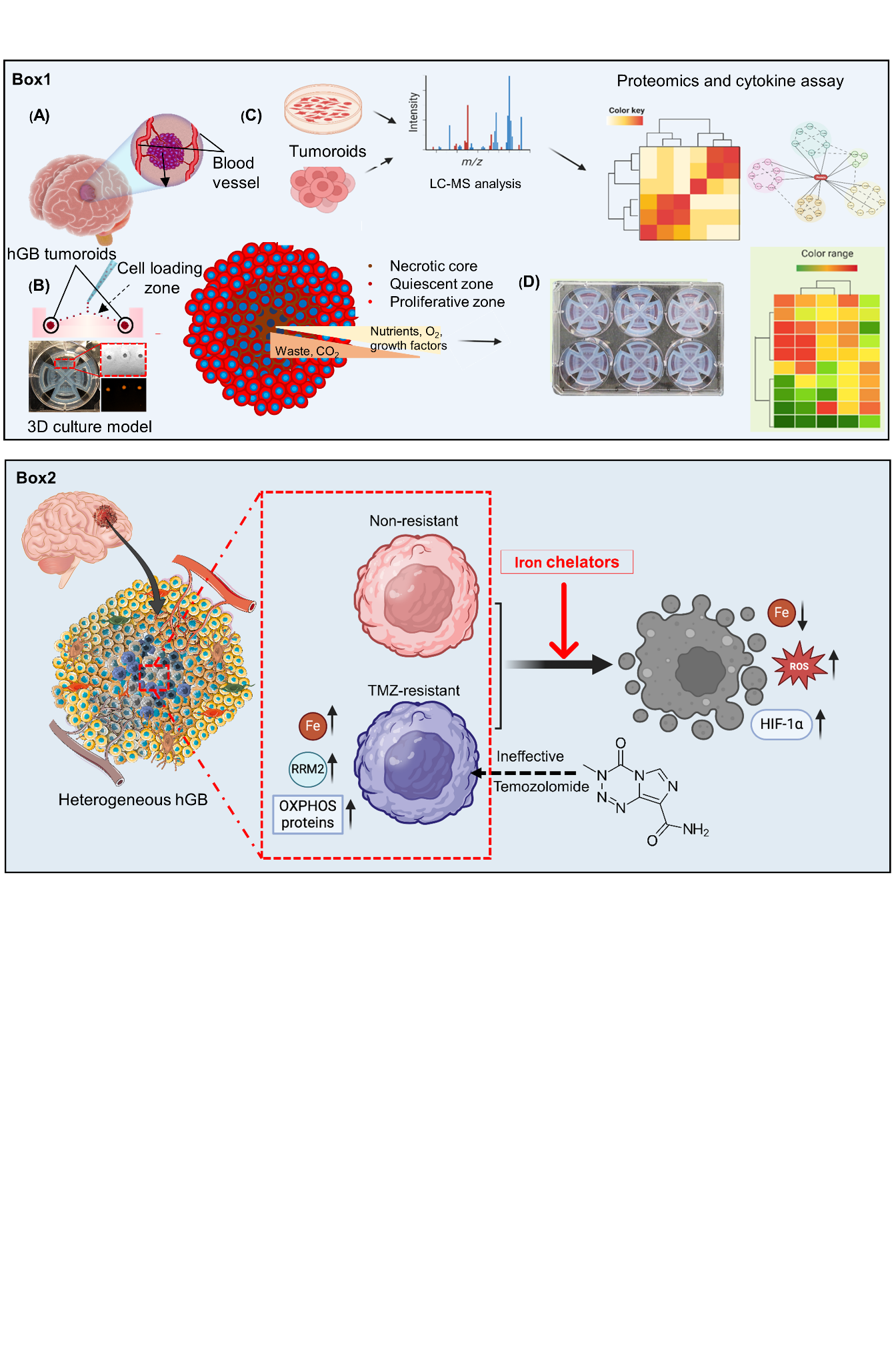


Live/dead imaging of U251 cells after four days encapsulation in hydrogel matrix (Fig. S2A) and confocal imaging of actin filaments visualized with phalloidin staining, comparing the invasion pattern of non-resistant vs. TMZ-resistant tumoroids. The non-resistant tumoroids developed a star-shaped invasion pattern with extended protrusions into the healthy areas of the matrix, resembling diffuse gliomas (Fig. S2B), verified the compatibility of the hydrogel. In contrast, TMZ-resistant tumoroids exhibited scattered tumor growth with dispersed protrusions into the surrounding matrix. A closer look at the invading cells disclosed that the non-resistant cells were elongated with clustered actin, finger-like projections into the surrounding TME, and filopodia protrusions, as seen in mesenchymal cells [50,51]. However, TMZ-resistant cells were rounded with bleb-like projections that are shallow and actin-rich, podosome protrusions, as seen in amoeboid cells. This migration plasticity confirms decreased adhesion properties in resistant cells related to mesenchymal to amoeboid transition (MAT), one of the stem cell characteristics [52-54].

TMZ-resistant cells with a half-maximal inhibitory concentration (IC50) of 674 µM, which was six times higher than the non-resistant cells (111 µM), were obtained (Fig. S2C). The IC50 for TMZ-resistant tumoroids was 5000 µM, whereas this value was 250 µM for non-resistant tumoroids (Fig. S2D). We evaluated the sensitivity of cells grown in 2D and 3D tumoroid cultures. To this aim, tumoroids (200 µm in diameter) were formed using TruSphere culture plate (Apricell Biotechnology, Fig. S2E) and were treated with a range of 0-5000 µM TMZ for up to four days. Both non-resistant and TMZ-resistant tumoroids displayed significantly lower chemosensitivity when compared to 2D cultures (Fig. S2F).


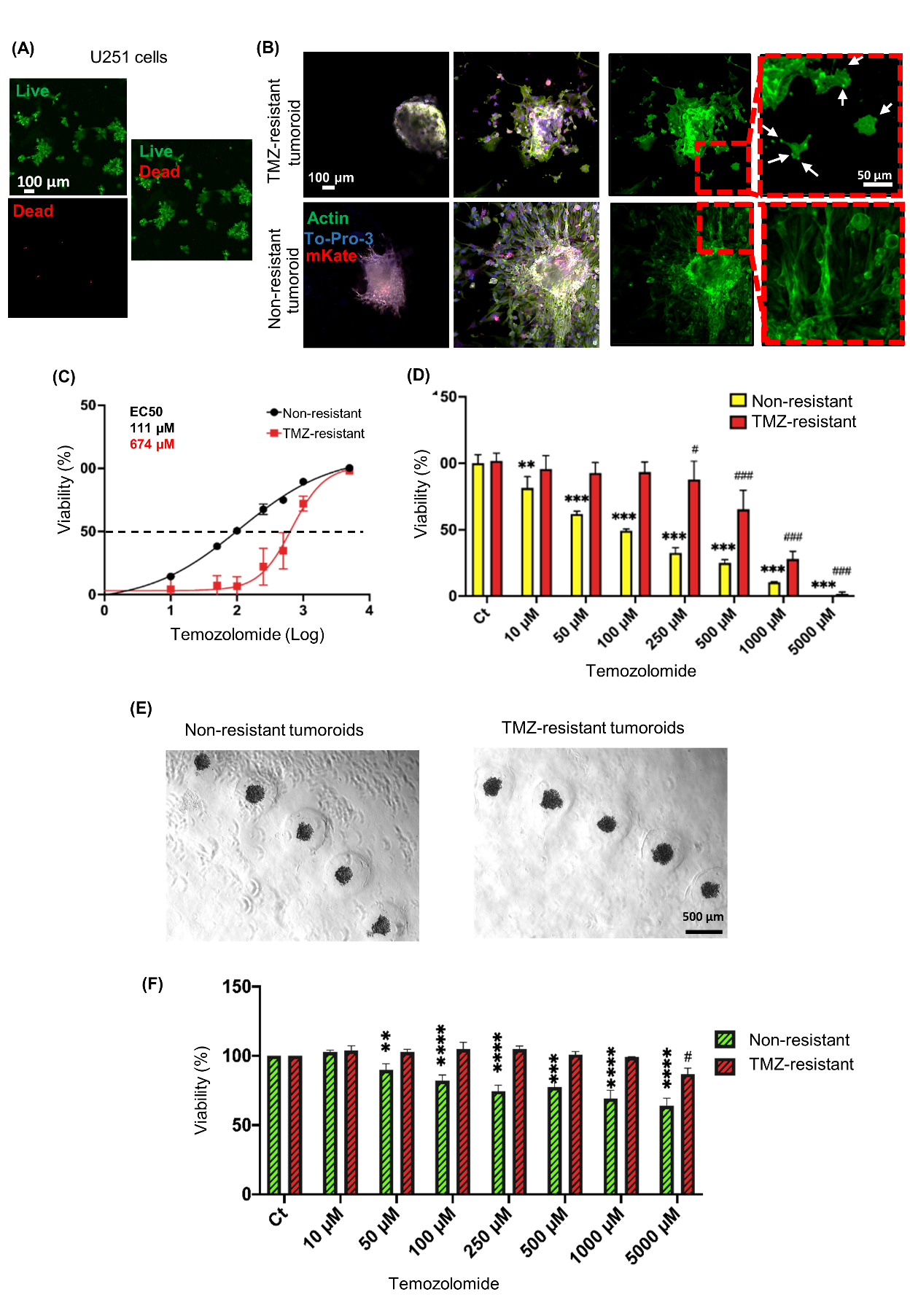


Day 0

Day 4

Day 4

Day 0

**Figure S2**. (A) Live/dead assay of U251 cells after four days of encapsulation in hydrogel matrix. (B) Confocal imaging of actin filaments, visualized with phalloidin staining, comparing the invasion pattern of non-resistant vs. TMZ-resistant tumoroids after four days encapsulation in hydrogel matrix. (C) EC50 of non-resistant and TMZ-resistant cells treated with TMZ. (D) Viability of non-resistant and TMZ-resistant cells treated with 0-5000 µM of TMZ for 96h. (E) Formation of non-resistant and TMZ-resistant tumoroids. (F) Viability assay of 3D non-resistant and TMZ-resistant tumoroids treated with 0-5000 µM of TMZ for 96h. N=3 biological independent experiment in (A). The values are mean ± SEM of N =15 biologically independent samples examined over three independent experiments in (D, F).

Scatter plots reveal the sequence converge percentage (SC%) in non-resistant and TMZ-resistant cells in 2D and 3D cultures (Fig. S3A). Notably, 87% and 20% of differentially regulated proteins showed SC < 50% and SC < 2%, respectively (Fig. S3A).

Volcano plot analysis revealed that BST2, FTL, FTH1, ASAH1, SERPINH1 and ISG15 were upregulated in TMZ-resistant cells (p<0.05); among which FTL and FTH1 have important role in iron metabolism, in line with the PCA results, and SERPINH1 and ISG15 were associated to cancer stemness. One of the most significantly downregulated proteins was glial fibrillary acidic protein (GFAP), a pan neuro-biomarker which indicates dedifferentiation in TMZ-resistant cells. GFAP’s expression can be influenced by hypoxia, providing further confirmation of its pronounced downregulation observed in TMZ-resistant tumoroids compared to other experimental groups (p<0.05) (Fig. S3B). In TMZ-resistant tumoroids, the most prominently overexpressed protein was ASS1, a key component in the oxidative phosphorylation pathway known to play a crucial role in drug resistance mechanisms. Conversely, Cathepsin L1 (CTSL) significantly decreased in TMZ-resistant tumoroids compared to their non-resistant counterparts (p<0.05). This finding suggests a reduction in lysosomal protein expression in the context of 3D cultures, further supported by the enriched KEGG pathway identified in cluster 1 (Fig. S3C).


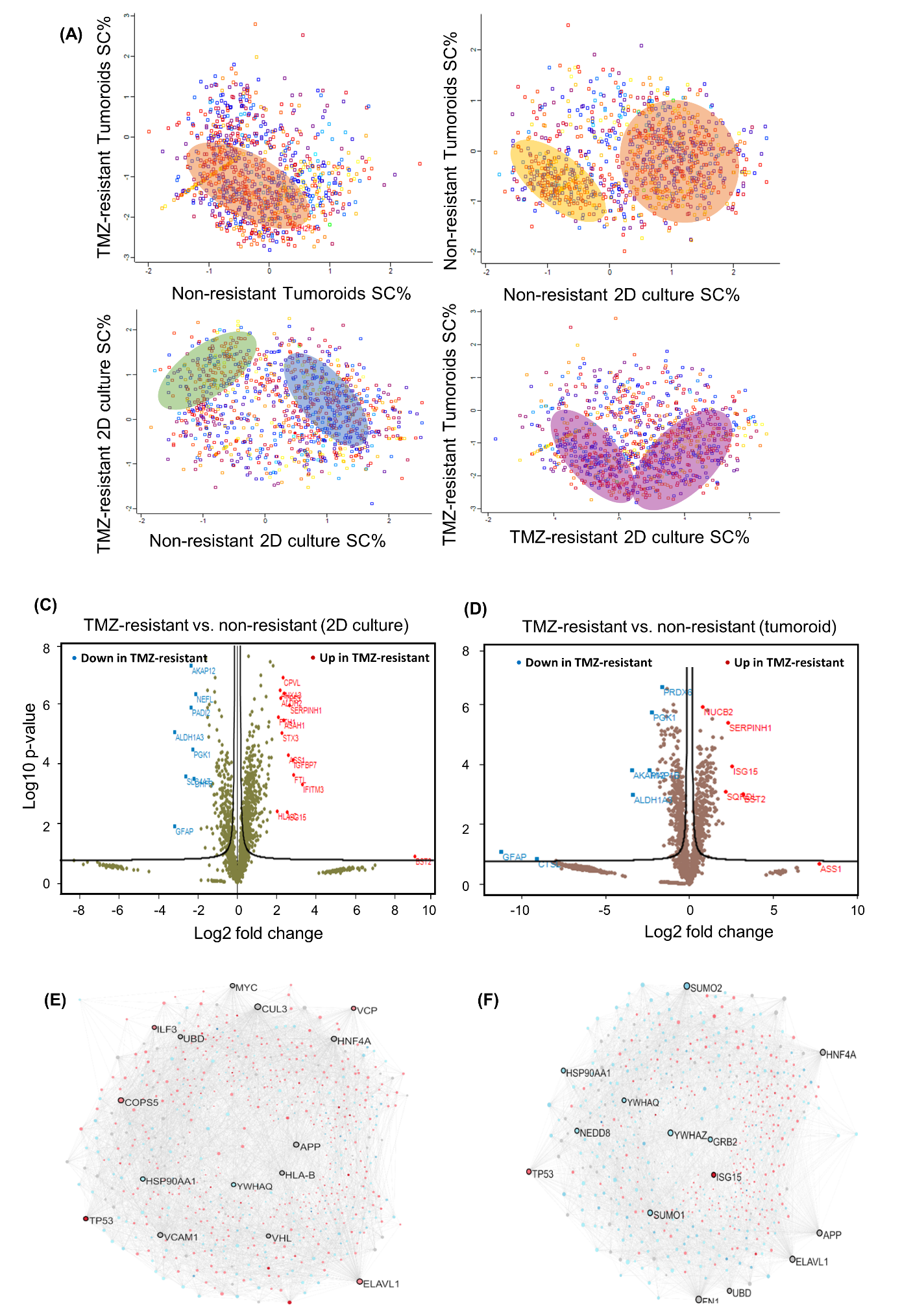


**(B)**

**(C)**

**(B)**

**(C)**

**Figure S3.** (A) Scatter plots for sequence converge percentage (SC%) in non-resistant and TMZ-resistant cells in 2D and 3D cultures. Volcano scatter plot of 2D (B) and 3D (C) non-resistant vs TMZ-resistant cells and tumoroids, respectively. The red or blue dots indicate proteins significantly (q < 0.05) upregulated (log2 fold change ≥ 0.5) or downregulated (log2 fold change ≤ −0.5) respectively. Proteins with more than 2-fold up or downregulation are annotated.


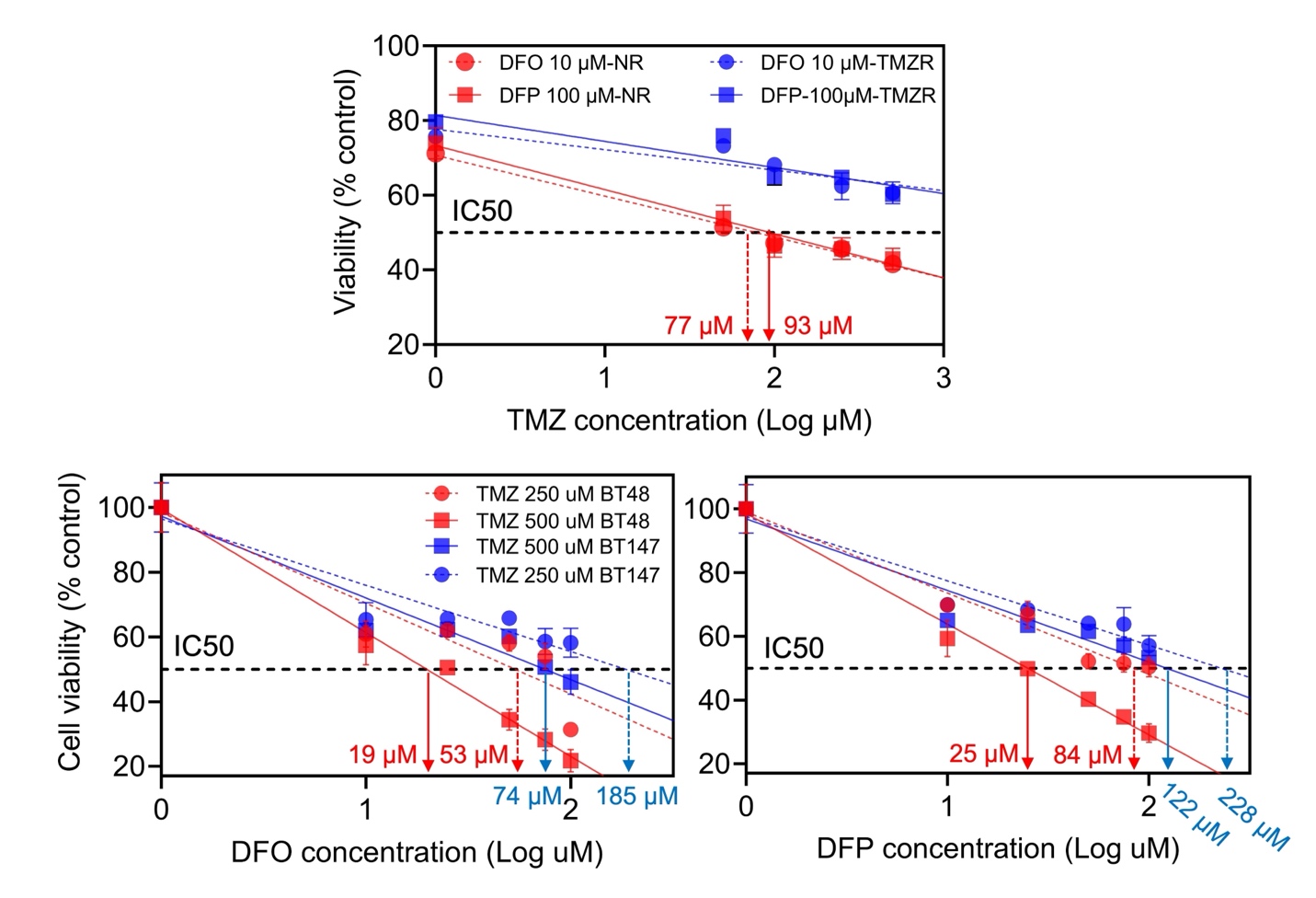


**Figure S4.** IC50 values for the co-treatment of non-resistant and TMZ-resistant U251 tumoroids with TMZ, DFO, and DFP are presented. (A) Synergistic effects were observed in non-resistant tumoroids with combinations of 10 µM DFO + 93 µM TMZ and 100 µM DFO + 77 µM TMZ. (B) Synergistic interactions between TMZ and either DFO (left) or DFP (right) were also evident in patient-derived GBM models, including newly diagnosed (BT48) and recurrent chemo-resistant (BT147) samples. The TMZ concentrations corresponding to IC50 values are indicated on the x-axis.

**(B)**

**(A)**


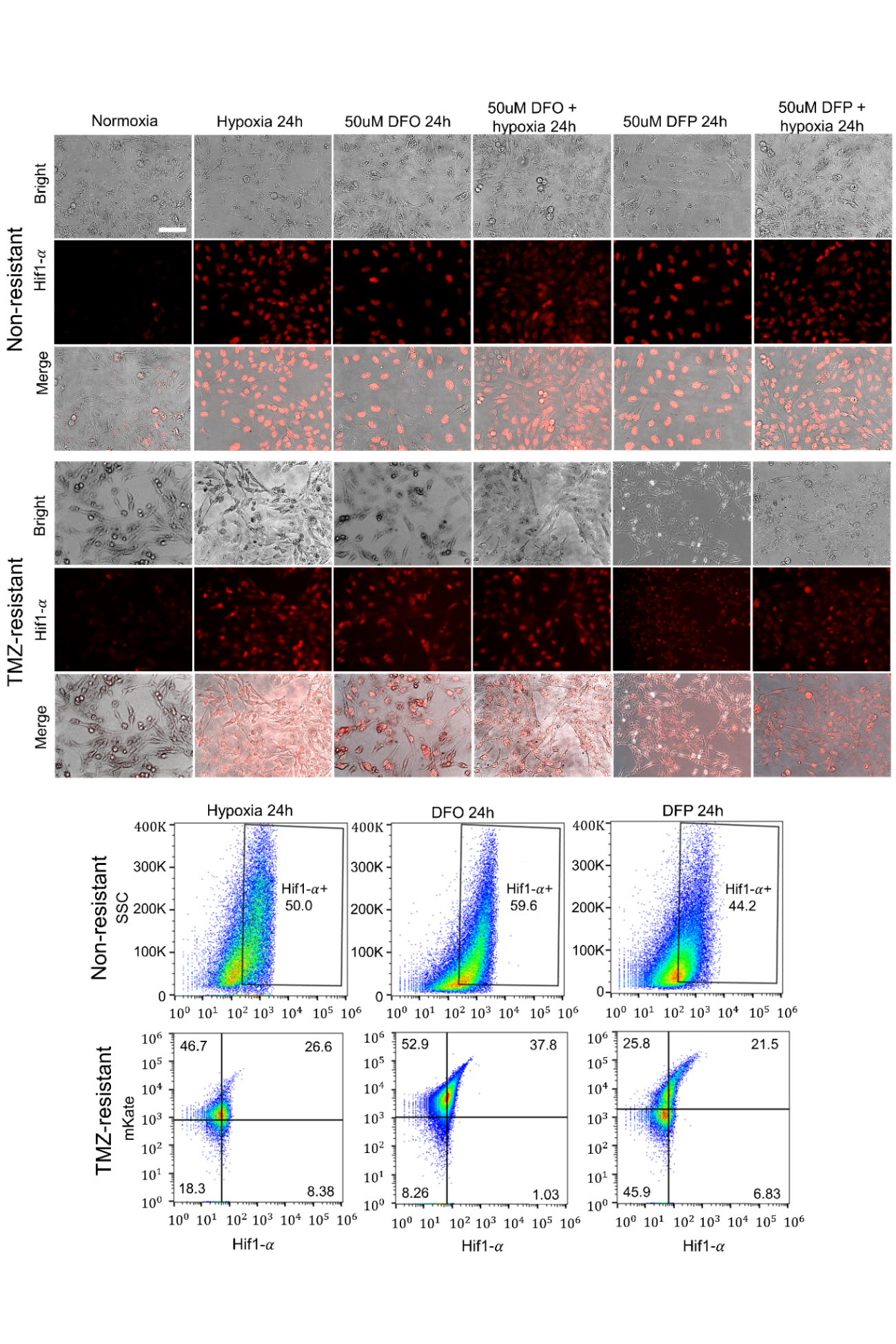


**(A)**

**(B)**

**Figure S5.** (A) Immunostaining of HIF1-α expression in U251 non-resistant and TMZ-resistant cells exposed to 24h of hypoxia, 50 mM DFO, 50 mM DFP, and combination of hypoxia and either DFO or DFP. (B) Semi-quantification of HIF1-α expression revealed that non-resistant cells exhibited higher accumulation of HIF1-α compared to TMZ-resistant cells in all treatment conditions. DFO treated non-resistant and TMZ-resistant cells expressed higher level of HIF1-α (%59, %37), respectively, compared to both DFP treatment (%40, %21) and hypoxia incubation (%50, 26%).


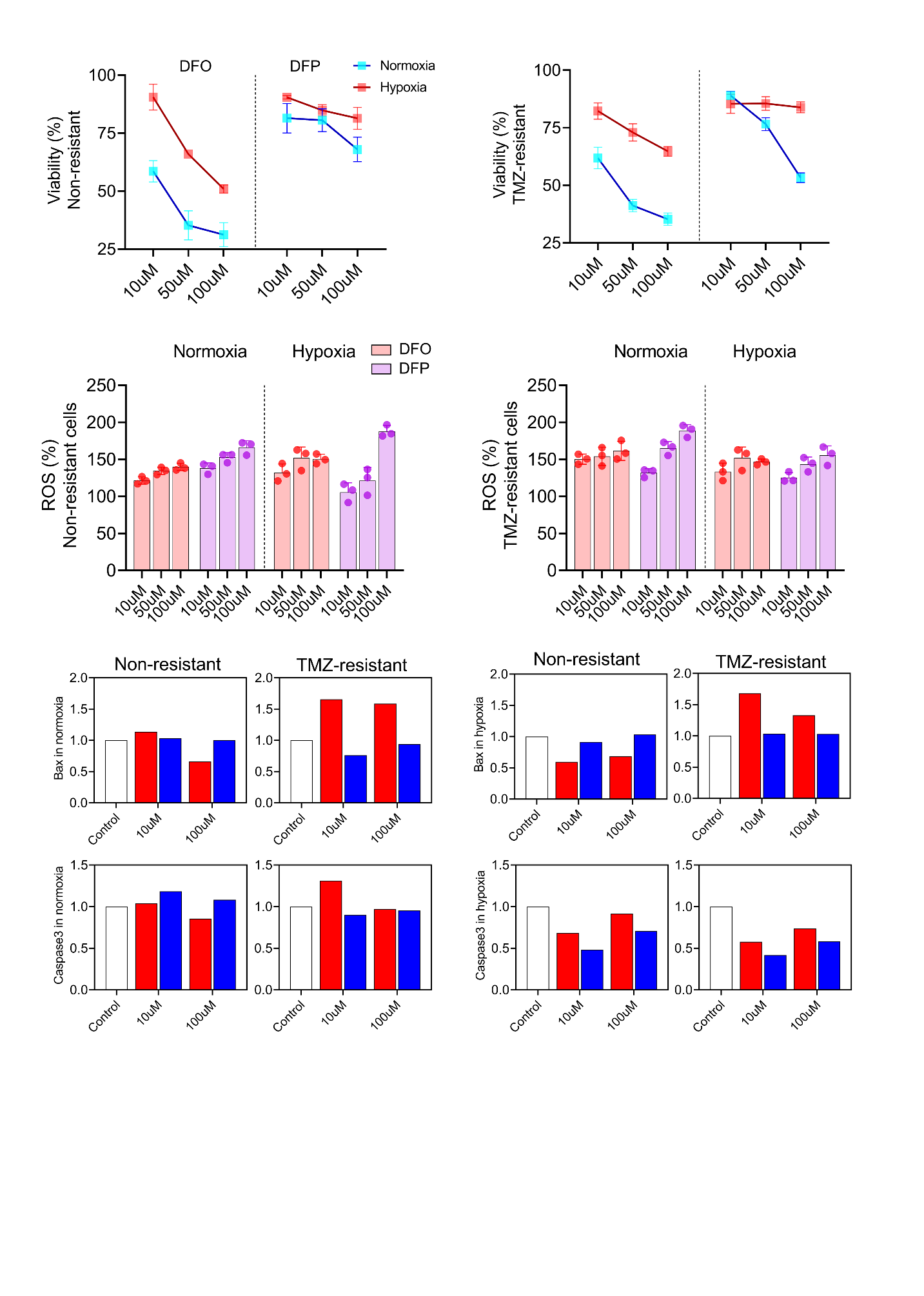


**Figure S6.** (A) Iron chelators reduced viability of non-resistant and TMZ-resistant cells after 72h, showing significant reduction in response to DFO but less sensitivity to DFP, with hypoxia counteracting the viability reduction. (B) Iron chelators induced ROS generation after 72h with hypoxia reducing the effect of DFP treatment in both non-resistant and TMZ-resistant cells. N=3 biological independent experiments and statistically significant at a P-value < 0.05. (C) Densitometry analysis of WB showed bax expression was increased only in response DFO in normoxic and hypoxic TMZ-resistant cells. However, bax was not increased in NR cells under any treatment. Although neither of the chelators showed significant increase in expression of caspase3, it was reduced in hypoxia.

**(A)**

**(B)**

**(C)**

**Figure S7.** Iron chelators DFO and DFP are highly selective in removing iron without affecting homeostatic levels of other metals. Intracellular levels of Zinc, Manganese, Magnesium, and copper were not significantly changed in GBM cells in response to 10-100 µM of DFO and DFP. The expression of calcium was upregulated monotonically with the dose of iron chelators. N=3 biological independent experiments and statistically significant at a P-value < 0.05.


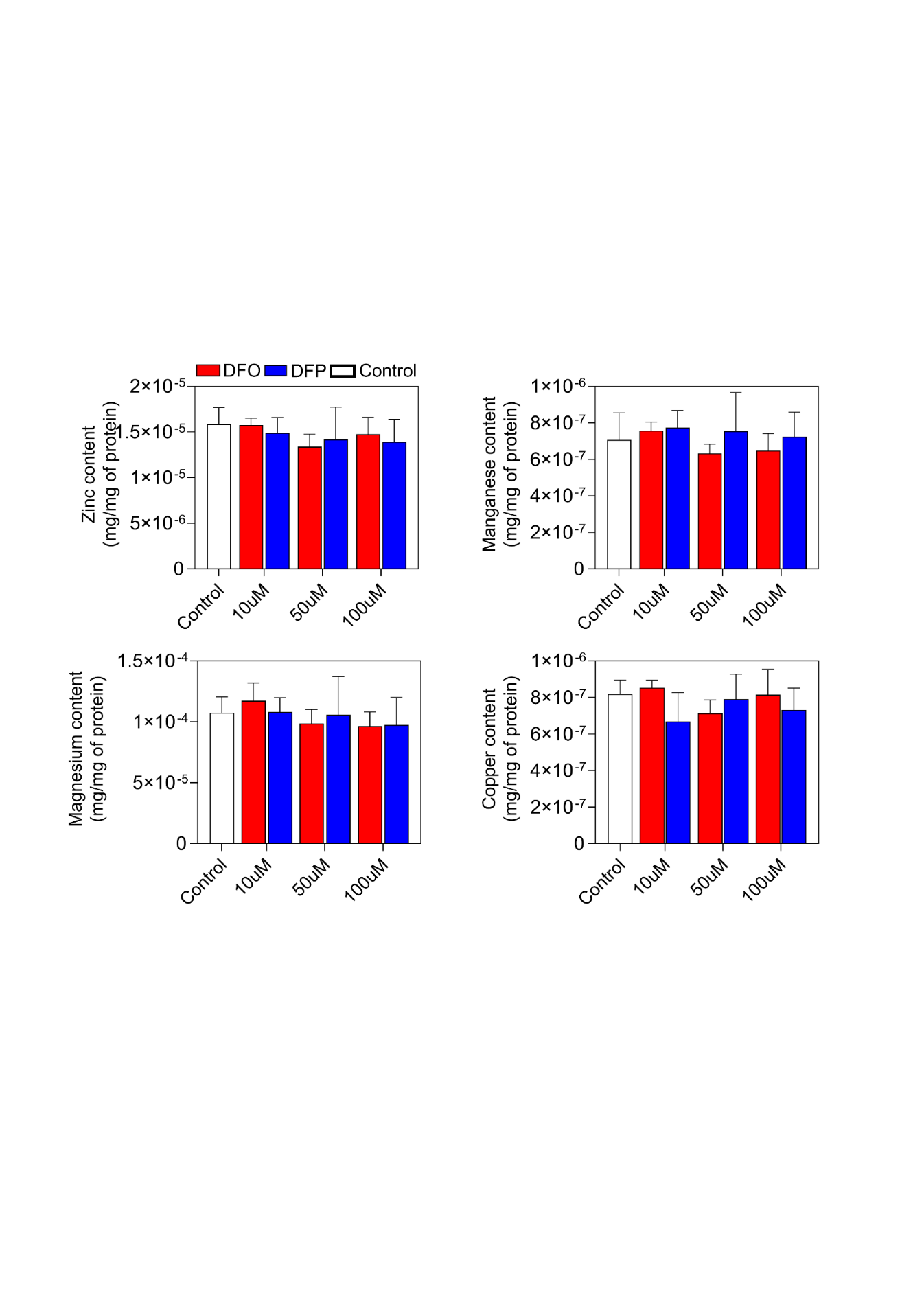

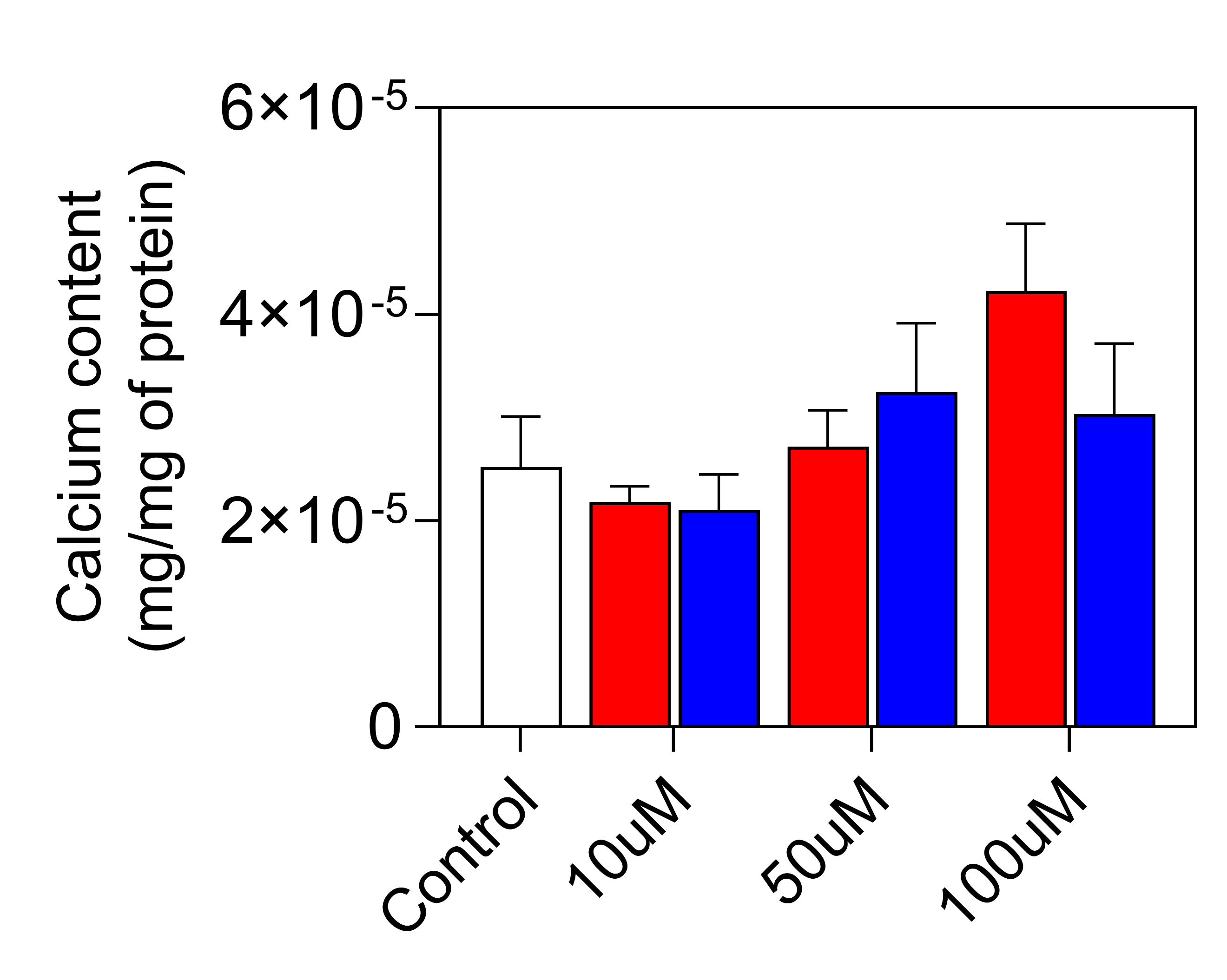

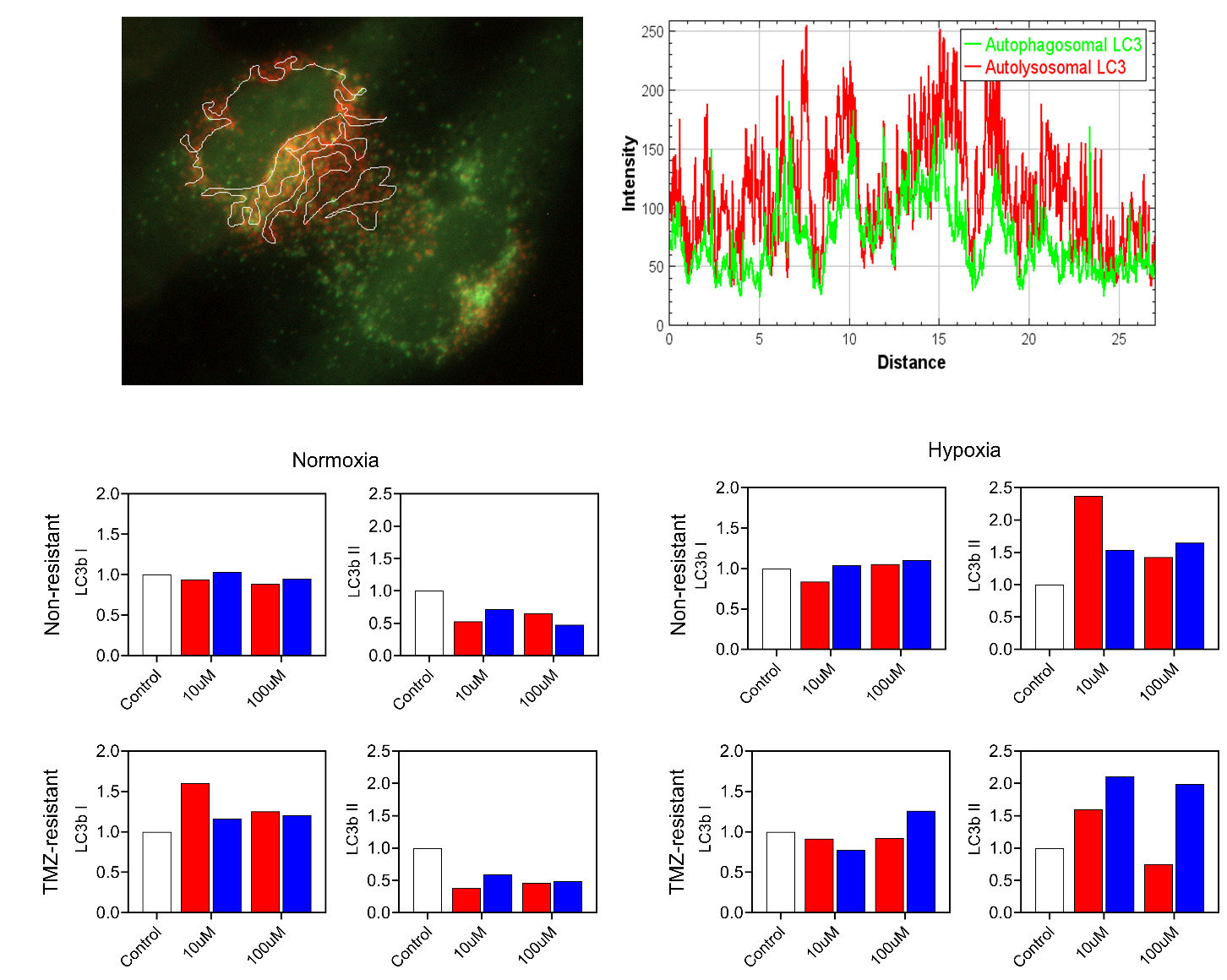


**(A)**

**(B)**

Intensity (a.u.)

Distance (μm)

**Figure S8**. (A) Intensity plot visualized peaks mismatch, i.e., autophagic flux, and peaks overlap, i.e., flux inhibition. The X-axis represents distance (μM) and the Y-axis represents intensity (arbitrary units, a.u.). (B) Densitometry analysis of WB showed that the ratio of LC3b-II to LC3b-I was increased in hypoxia compared to normoxia for both non-resistant and TMZ-resistant cells in response to DFO and DFP treatment, confirming the regulation of autophagic flux by iron chelators in hypoxia. Scale bar is 20 μm.


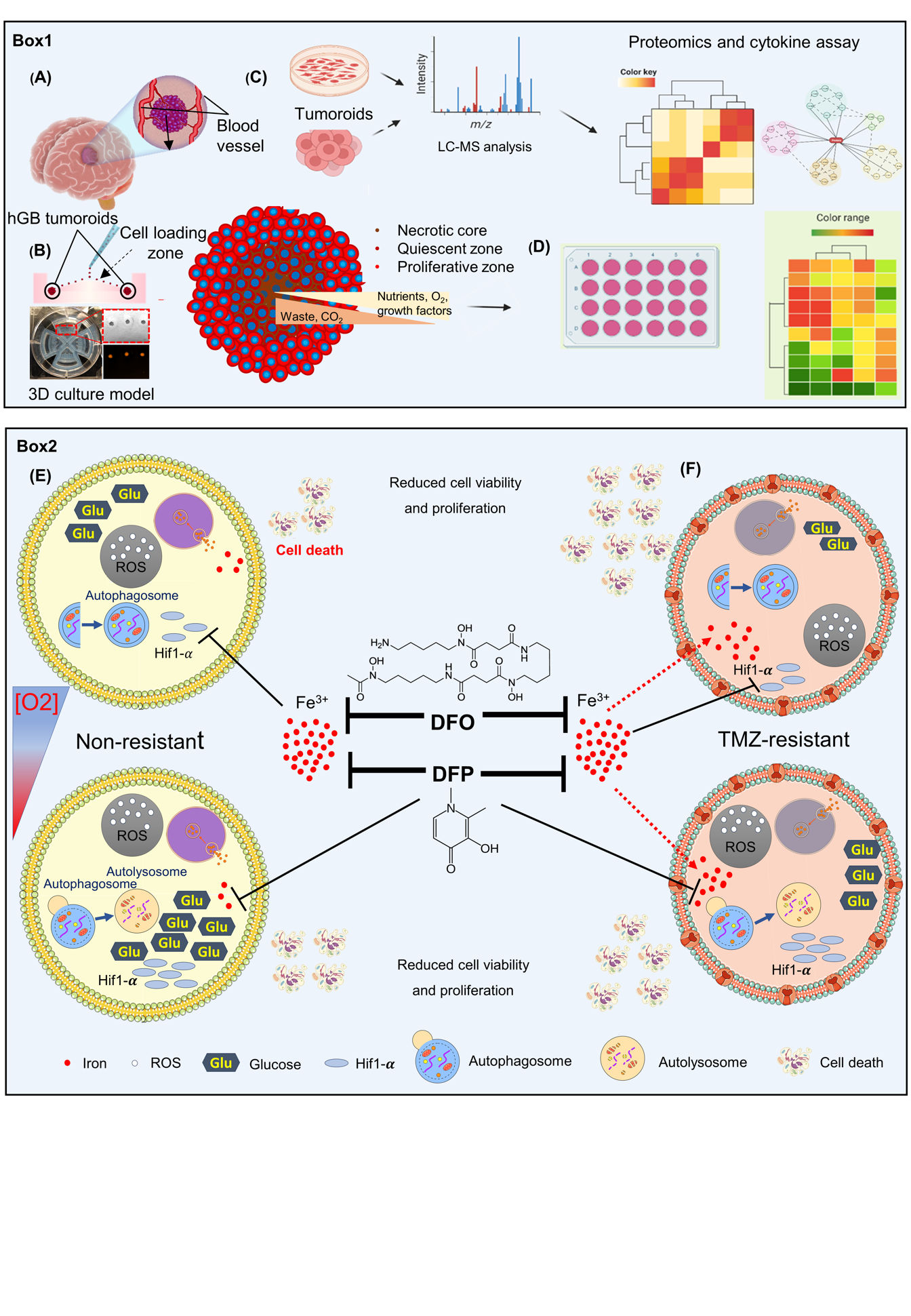


**(A)**

**(B)**

**Figure S9**. Downstream variation induced by iron and oxygen deficiency. Iron deficiency by DFO and DFP induces variations in HIF-1$\alpha$ expression, ROS generation, autophagic flux, viability, proliferation, and cell death, in hGBM non-resistant (A), and TMZ-resistant (B) normoxic (top) and hypoxic (bottom) cells. Graph prepared in Biorender.

**Figure S10.** Original uncropped western blots for the expression of HIF1-α, Bax, and β-actin in response to (A) DFO and (B) DFP, and the expression of Caspase 3 and LC3b in response to (C) DFO and (D) DFP, in non-resistant and TMZ-resistant cells in normoxia and hypoxia.
